# Live Imaging of Innate Immune Cell Sensing of Transformed Cells in Zebrafish Larvae: Parallels between Tumor Initiation and Wound Inflammation

**DOI:** 10.1371/journal.pbio.1000562

**Published:** 2010-12-14

**Authors:** Yi Feng, Cristina Santoriello, Marina Mione, Adam Hurlstone, Paul Martin

**Affiliations:** 1 Department of Biochemistry, School of Medical Sciences, University of Bristol, Bristol, United Kingdom; 2 Department of Physiology and Pharmacology, School of Medical Sciences, University of Bristol, Bristol, United Kingdom; 3 IFOM, the FIRC Institute of Molecular Oncology Foundation, Milan, Italy; 4 Faculty of Life Sciences, The University of Manchester, Manchester, United Kingdom; Fred Hutchinson Cancer Research Center, United States of America

## Abstract

It has not previously been possible to live image the earliest interactions between the host environment and oncogene-transformed cells as they initiate formation of cancers within an organism. Here we take advantage of the translucency of zebrafish larvae to observe the host innate immune cell response as oncogene-transformed melanoblasts and goblet cells multiply within the larval skin. Our studies indicate activation of leukocytes at very early stages in larvae carrying a transformed cell burden. Locally, we see recruitment of neutrophils and macrophages by 48 h post-fertilization, when transformed cells are still only singletons or doublets, and soon after this we see intimate associations between immune and transformed cells and frequent examples of cytoplasmic tethers linking the two cell types, as well as engulfment of transformed cells by both neutrophils and macrophages. We show that a major component of the signal drawing inflammatory cells to oncogenic HRAS^G12V^-transformed cells is H_2_O_2_, which is also a key damage cue responsible for recruiting neutrophils to a wound. Our short-term blocking experiments show that preventing recruitment of immune cells at these early stages results in reduced growth of transformed cell clones and suggests that immune cells may provide a source of trophic support to the transformed cells just as they do at a site of tissue repair. These parallels between the inflammatory responses to transformed cells and to wounds reinforce the suggestion by others that cancers resemble non-healing wounds.

## Abstract 2

Live imaging and genetic studies of the initial interactions between leukocytes and transformed cells in zebrafish larvae indicate an attractant role for H2O2 and suggest that blocking these early interactions reduces expansion of transformed cell clones.

## Author Summary

The translucency of zebrafish larvae allows us to live image the earliest dynamic interactions between host innate immune cells and oncogene-transformed cell clones as they first establish themselves as the precursors of full-blown cancer. These early associations manifest via cytoplasmic tethers between an immune cell and a transformed cell, and occasional phagocytic engulfment. Immune cells are first attracted to transformed cells at surprisingly early stages, before transformed cells have had a chance to form clones and are thus still singletons or doublets. We show that the key attractant is hydrogen peroxide (H_2_O_2_), which was also recently shown to be the essential early damage signal responsible for drawing neutrophils to wounds. Tissue transplantation experiments allow us to test which cells are responsible for generating the H_2_O_2_ attractant, and we show that both transformed cells and their otherwise healthy neighbors contribute. Blocking H_2_O_2_ synthesis, either pharmacologically or by morpholino-mediated knockdown of DUOX, the enzyme responsible for H_2_O_2_ synthesis in larval skin, very significantly reduces the numbers of neutrophils and macrophages drawn to transformed cell clones, and this results in reduced numbers of transformed cells, suggesting that innate immune cells play a trophic and/or support role in early transformed cell growth.

## Introduction

Cancers originate from one or a few clones of transformed cells that gain a growth advantage over neighboring normal cells, which, in turn, enables them to invade the host microenvironment and form a tumor [1],[2]. Decades of research using various murine tumor models, as well as analysis of human clinical tumor samples, has revealed how activation of various oncogenes and/or loss of tumor suppressor gene function, can intrinsically confer a growth advantage on transformed cells [1],[2]. However, it is now clear that many host-derived cellular and molecular components can also influence this cellular transformation [3]–[5]. In particular, there is considerable evidence that the host immune system plays a pivotal role in several conflicting aspects of cancer initiation and progression, both as a key player in immune elimination, to “find and destroy” transformed cells [6]–[8], and as an active assistant that may aid expansion and metastatic spread of a tumor [4],[9],[10].

Inflammation is a crucial function of the innate immune system that protects host tissues against dangerous insults that are detrimental to tissue homeostasis, including wound damage and pathogen invasion [11]. Acute inflammation, as triggered by wounding, is a rapid and self-limiting process: chemical mediators are induced in a tightly regulated sequence, and innate immune cells move in and out of the affected area, destroying infectious agents, and delivering growth factors and other signals that aid in repairing the damaged tissue [12]. However, when innate immunity goes awry, inflammation does not always resolve, and it is believed that chronic, “smouldering,” and often subclinical inflammation can be the root cause of many human pathologies, including cancer [13]–[16]. Because of difficulties in predicting when and where transformed cells may arise in an organism, very little is currently known about the earliest events whereby host tissues respond to somatic cell transformation prior to the emergence of any sign of malignant progression. When does the host first recognize transformed cells, and how do they interact? Answering this question begs a model that allows easy live, in vivo observation of these events.

Recently, the zebrafish, *Dano rerio*, has emerged as a new model organism in studies of cancer biology, with many molecular and cellular components that operate during tumorigenesis in mammals seemingly conserved in fish [17]–[19]. The translucency of zebrafish larvae makes it possible for us to observe how the transformed cells interact with various cellular components of their host environment in vivo, and at high resolution. During zebrafish development, innate immune cell types emerge as early as 15 h post-fertilization (hpf) [20],[21], and become competent to respond to infections and wounds from around 22 hpf [22],[23]. However, it is believed that mature adaptive immune cells emerge significantly later, not before at least 1 wk of larval life [24],[25]. This provides a further advantage, since it enables us to tease apart the contribution solely from innate immune cells to the earliest events of tumorigenesis, without complications from cross talk with the adaptive immune system.

In this study we have used oncogenic forms of Ras and Src to induce transformation of cells that reside within the larval skin. Our in vivo, live imaging movies indicate that these transformed cells activate and recruit host leukocytes from very early stages of development. We show that this initial recruitment of leukocytes to transformed cells is, at least in part, triggered by local synthesis of H_2_O_2_. This parallels the early damage signals that first draw innate immune cells towards wounds [26],[27]. However, instead of subsequent shutdown and resolution of the inflammatory response, as occurs following the acute wound response, the inflammatory response to transformed cells appears to amplify and progress towards that of a chronic inflammatory state as seen in chronic non-healing wounds. Inhibiting the generation of H_2_O_2_ prevents leukocyte recruitment towards wounds in zebrafish larvae, and we have found that it also prevents leukocyte recruitment towards transformed cells. Inhibition of leukocyte recruitment leads to a reduction in the growth of neoplastic cells, indicating an early trophic function for innate immune cells. Together, our data indicate homologies, at both the cellular and molecular levels, between the transformed-cell-induced host innate immune response and a wound inflammatory response.

## Results

It has previously been reported that oncogenic forms of Ras induce malignant transformation of various cell types in zebrafish larvae, and lead to tumor formation in adults [28]–[30]. A zebrafish melanoma model has previously been established in which the melanocyte-specific promoter *microphthalmia-associated transcription factor a* (*mitfa*) is used to drive oncogenic human HRas (HRas^G12V^, hereafter referred to as V12RAS) expression in melanocytes [29]. To aid easy identification and live imaging studies of transformed cells, this mitfa:V12RAS transgene construct also contains a *mitfa*-promoter-driven mCherry expression cassette. G_0_-injected animals develop ectopic melanocyte clones during the larval stages, [29] and, until the larvae become heavily pigmented, we can live image the interactions of the transformed melanocytes with the host environment. In this model, tumor nodules often form at sites where there is likely to have been local tissue damage, for example, the female pelvic fin (unpublished observation). Expression profiling studies carried out in this V12RAS-induced melanoma model reveal extensive up-regulation of pro-inflammatory genes, for example, *ifn1*, *il8*, and *irak3*, in the tumor tissue compared with control tissue (P. Walker, M. Jones, S. He, C. Michailidou, N. Haud, et al., unpublished data), suggesting that inflammation may play a role during V12RAS-induced tumorigenesis.

### The Presence of Transformed Cells Activates Leukocytes in Zebrafish Larvae

**Figure 1:**
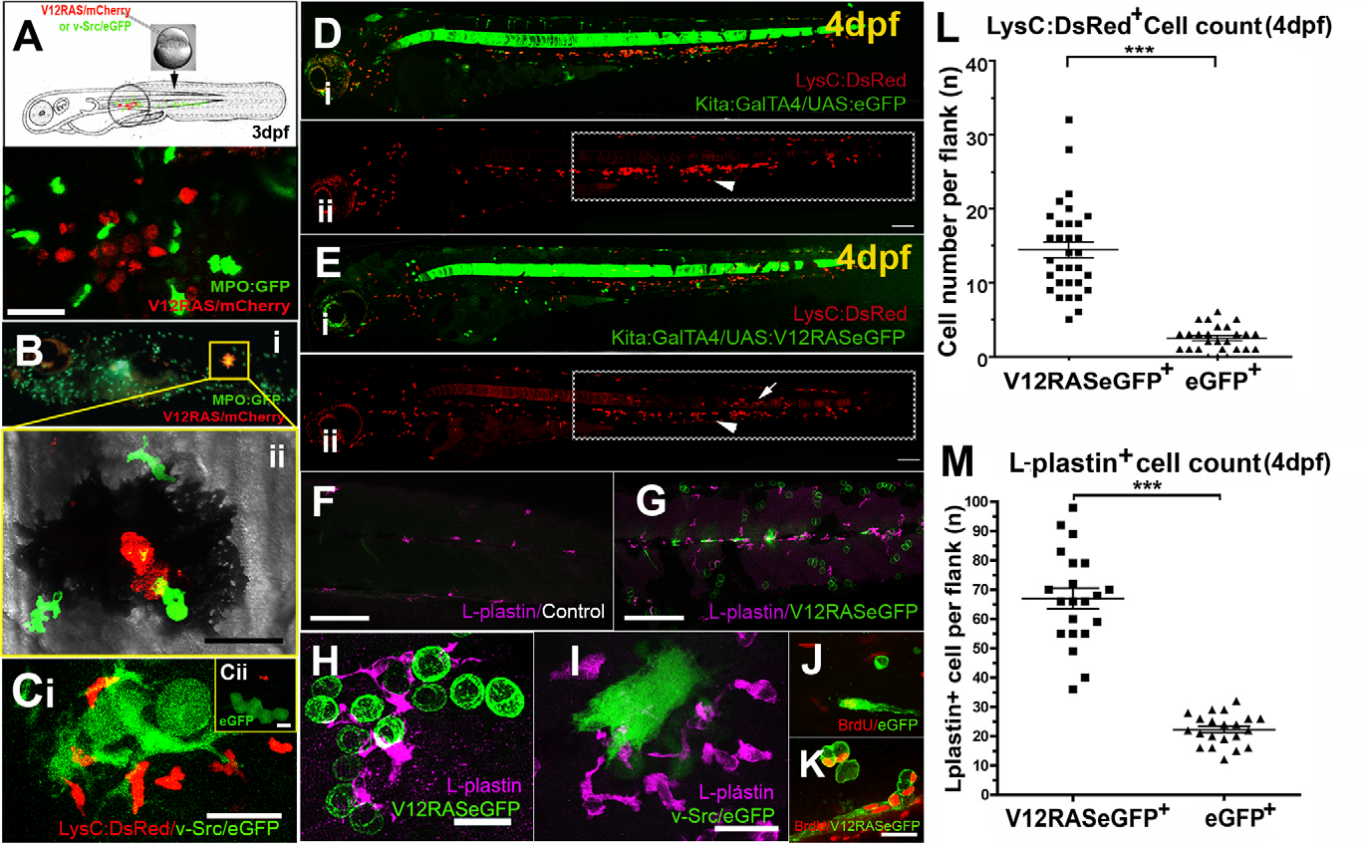
Activation of leukocytes in zebrafish larvae by oncogene-transformed cells. (A) Schematic of the procedure for transient induction of V12RAS/v-Src in embryos that also have fluorescently tagged neutrophils, with an example of a V12RAS^+^ melanoblast clone (red) in a 3-dpf larva also expressing eGFP (green) in neutrophils. (B) (i) A 5-dpf MPO:GFP larva with a V12RAS^+^ clone (red). (ii) High-magnification view of the inset in (i), which is a single image from a time-lapse movie (Video S1A) of GFP-tagged neutrophils actively interacting with a V12RAS^+^ clone; note that most of the red fluorescent signal is quenched by melanocyte pigment. (C) (i) A single image from a time-lapse movie showing LysC:DsRed^+^ cells recruited by v-Src^+^ (green) cells in a 3-dpf larva (Video S2B). (ii) An equivalent image from a time-lapse movie showing no recruitment of LysC:DsRed^+^ cells to GAP43-eGFP-expressing cells in a control larva (Video S2A). (D) (i) Low-magnification, two-channel, lateral view of a control *Tg (kita:GalTA4, UAS:eGFP, LysC:DsRed)* larva at 4 dpf. (ii) Single-channel view to highlight only the DsRed-tagged leukocytes. The box highlights these cells located within the hematopoietic tissue. Arrowhead indicates LysC:DsRed^+^ cells in the hematopoietic tissue. (E) As for (D) but of a *Tg (kita:GalTA4, UAS:V12RASeGFP, LysC:DsRed)* larva at 4 dpf. The boxed zone in (ii) indicates how the LysC:DsRed^+^ cells have largely dispersed from the hematopoietic tissue into the flank skin. Arrowhead indicates LysC:DsRed^+^ cells in the hematopoietic tissue; arrow indicates LysC:DsRed^+^ cells in the skin tissue. (F) A confocal *Z*-stack projection of the flank of a control larva in the trunk region. (G) Equivalent image to (F) but of a V12RAS^+^ larva. Both are stained with the anti-L-plastin antibody (magenta). (H) A high-magnification view of a larva similar to that in (G), illustrating the association of L-plastin^+^ cells (magenta) with V12RAS^+^ cells (green). (I) A similar larvae to that in (H), but with v-Src-expressing cells (green). (J) Anti-BrdU (red) immunostaining of control mucus-secreting cells (green). (K) Anti-BrdU (Red) immunostaining of V12RAS^+^ mucus-secreting cells (green). (L) Quantification of numbers of LysC:DsRed^+^ cells present in the skin of the trunk in the region indicated by boxes in (D) and (E). (M) Quantification of the number of L-plastin^+^ cells present in the trunk epidermis in regions shown in (F) and (G). ***, *p*<0.001. Scale bars: (A), 48 µm; (B,C), 24 µm; (D–G), 150 µm; (H–K), 20 µm.

To investigate potential early interactions between transformed cells and the host immune system, we used transgenic zebrafish lines that have fluorescently labeled leukocytes. Several such zebrafish lines have now been generated and characterized [31]–[33]. We first injected the mitfa:V12RAS-mitfa:mCherry construct into one-cell-stage *Tg(BACmpo:eGFP)^i114^* (hereafter referred to as MPO:GFP) embryos with eGFP-tagged neutrophils [31], and followed sporadic V12RAS expression in melanocytes and their precursors, melanoblasts. We observed a significant enrichment of eGFP^+^ cells adjacent to mCherry^+^ transformed cells (26/28 larvae with ectopic clones) (Figure 1A) at 4 dpf. A similar association was not seen after injection of mitfa:mCherry control plasmid (Video S1C). Our live imaging of V12RAS^+^mCherry^+^ melanocyte clones in MPO:GFP larvae revealed that MPO^+^ cells mount an active inflammatory response towards even very few of these V12RAS^+^ cells and are able to engulf fragments of them (Figure 1B; Video S1A). These data indicate that V12RAS-transformed melanocytes induce a host inflammatory response from the very earliest stages. In order to address whether such an inflammatory response is unique to RAS-induced transformation, or whether this is a more general host response to oncogene-induced cell transformations, we have used a v-Src model that has recently been used to study the interaction of transformed cells and their normal epithelial neighbors in zebrafish larval skin [34]. We use the same pBR-Tol2-UAS-GAP43-GFP-SC-v-Src construct to induce clones of v-Src-transformed cells that co-express GAP43-eGFP in *Tg(LysC:DsRed)* embryos. All the resulting embryos that have clones of v-Src over-expressing cells show active recruitment of leukocytes to these clones, both when live imaged to visualize neutrophil attraction (Figure 1Ci; Video S2B), and in fixed specimens immunostained with an antibody against the pan-leukocyte marker, L-plastin (Figure 1I), which reveals all leukocytes. Again, there is no such recruitment of immune cells to fluorescent cells after injection of GAP43-eGFP control plasmid (Figure 1Cii; Video S2A).

Next, we wanted to determine whether this response extended beyond the melanocyte lineage. We continued to focus on skin as a model tissue by studying expression of V12RAS^+^ cells in another cell population that resides in the epidermis and asked whether these cells too could trigger a similar innate immune response. The zebrafish *kita* promoter drives gene expression in melanocytes and a subpopulation of mucus-secreting cells of zebrafish larvae from 24 hpf (*kita* expression pattern [35]). In zebrafish skin, mucus-secreting cells are scattered throughout the epidermis as individuals and are analogous to goblet cells that have the potential to give rise to rare carcinoid tumors in humans [36]. In the double transgenic line generated by crossing the *Et*(*kita:GalTA4,UAS:mCherry*)*hzm1* (hereafter referred to as kita:GalTA4) driver line [37] with the *Tg*(*UAS:eGFP-H-RASV12*)*io6* reporter line (hereafter referred to as UAS:V12RASeGFP) [38], larval offspring express oncogenic V12RASeGFP fusion protein in kita-expressing cells. The membrane localization of the V12RASeGFP fusion protein provided significant optical advantages for our subsequent live imaging studies.

Mucus-secreting cells expressing V12RAS exhibit abnormal overgrowth, as indicated by increased BrdU incorporation, in comparison to control eGFP-expressing cells (Figure 1J and 1K) and gradually form multicellular clumps within the epidermis of the larvae. To examine how host leukocytes react to these transformed cells, we crossed the *Tg(kita:Gal4, UAS:V12RASeGFP)* double transgenic fish (hereafter referred to as the V12RAS line) with *Tg(LysC:DsRed)* [33] fish to obtain embryos in which lysozyme C positive (LysC^+^) leukoctyes are labeled by DsRed (red) and V12RAS-transformed cells are labeled by eGFP (green). *Tg(kita:GalTA4, UAS:eGFP)* fish (hereafter referred to as the eGFP line) were crossed with *Tg(LysC:DsRed)* fish to obtain control larvae that have eGFP-labeled, but otherwise normal, goblet cells, as well as DsRed-labeled LysC^+^ cells. The live images of control eGFP larvae versus V12RAS^+^ larvae at 4 dpf (Figure 1D and 1E) show that there are increased numbers of LysC:DsRed^+^ cells distributed within the trunk skin of larvae with transformed cells (Figure 1D, 1E [dotted box], and 1L), indicating that leukocytes are activated by the presence of V12RAS^+^ cells in the epidermis.

To assess the responsiveness of all of the host innate immune cells, beyond just those that are LysC^+^ (largely neutrophils), we stained larvae with an antibody against L-plastin, and, again, we observed a marked increase in recruited cells, this time including not only neutrophils but also macrophages, into the skin of the trunk region (Figure 1F, 1G, and 1M). Moreover, we found that leukocytes were often associated with V12RAS^+^ cells in these larvae (Figure 1H). A study in *Drosophila* has reported that tumors can systemically stimulate proliferation of the host hematopoietic lineage [39]. To determine whether this is the case in our zebrafish model with the presence of V12RAS^+^ cells, we counted the number of LysC:DsRed^+^ cells in V12RAS^+^ larvae compared with in their V12RAS^+^ siblings by fluorescence-activated cell sorting and found no overall increase of LysC^+^ cells in V12RAS^+^ larvae (data not shown). However, we did see reduced numbers of LysC:DsRed^+^ cells in the hematopoietic tissue in V12RAS^+^ larvae (Figure 1Eii), where most larval LysC^+^ cells normally reside at this developmental stage (Figure 1Dii) [33], suggesting that these cells are drawn to the transformed cells in the skin. Together these observations indicate that V12RAS^+^ transformed cells induce an inflammatory environment that draws leukocytes to the skin and, moreover, that transformed cells also direct local recruitment of leukocytes towards them. To determine whether this inflammatory response is reflected by up-regulation of pro-inflammatory markers, we performed reverse transcription PCR (RT-PCR) on larvae carrying a transformed goblet cell burden, and indeed we see increased levels of *tnf*α, *cxcl1*, *il8*, *ifn1*, and *il1*β in these larvae (Figure S1A). Moreover, if we look by quantitative PCR (qPCR) at levels of two of these genes, *il1*β and *cxcl1*, we see their clear induction in larvae where V12RAS expression is induced by heat shock only 6 h before RNA extraction, suggesting that pro-inflammatory gene expression is a rapid response to V12RAS expression (Figure S1B).

### V12RAS-Driven Leukocyte Recruitment in Larval Skin Is Analogous to That Triggered by a Wound

It is well established that tissue damage induces an inflammatory response and that innate immune cells are rapidly recruited to any wound site [12]. Live imaging studies using leukocyte reporter transgenic larval zebrafish have enabled detailed cell behavioral analysis of this in vivo recruitment of innate immune cells to wounds [32],[40], as well as the subsequent resolution of these cells, which occurs over a similar time course to that seen in mammalian systems.

**Figure 2:**
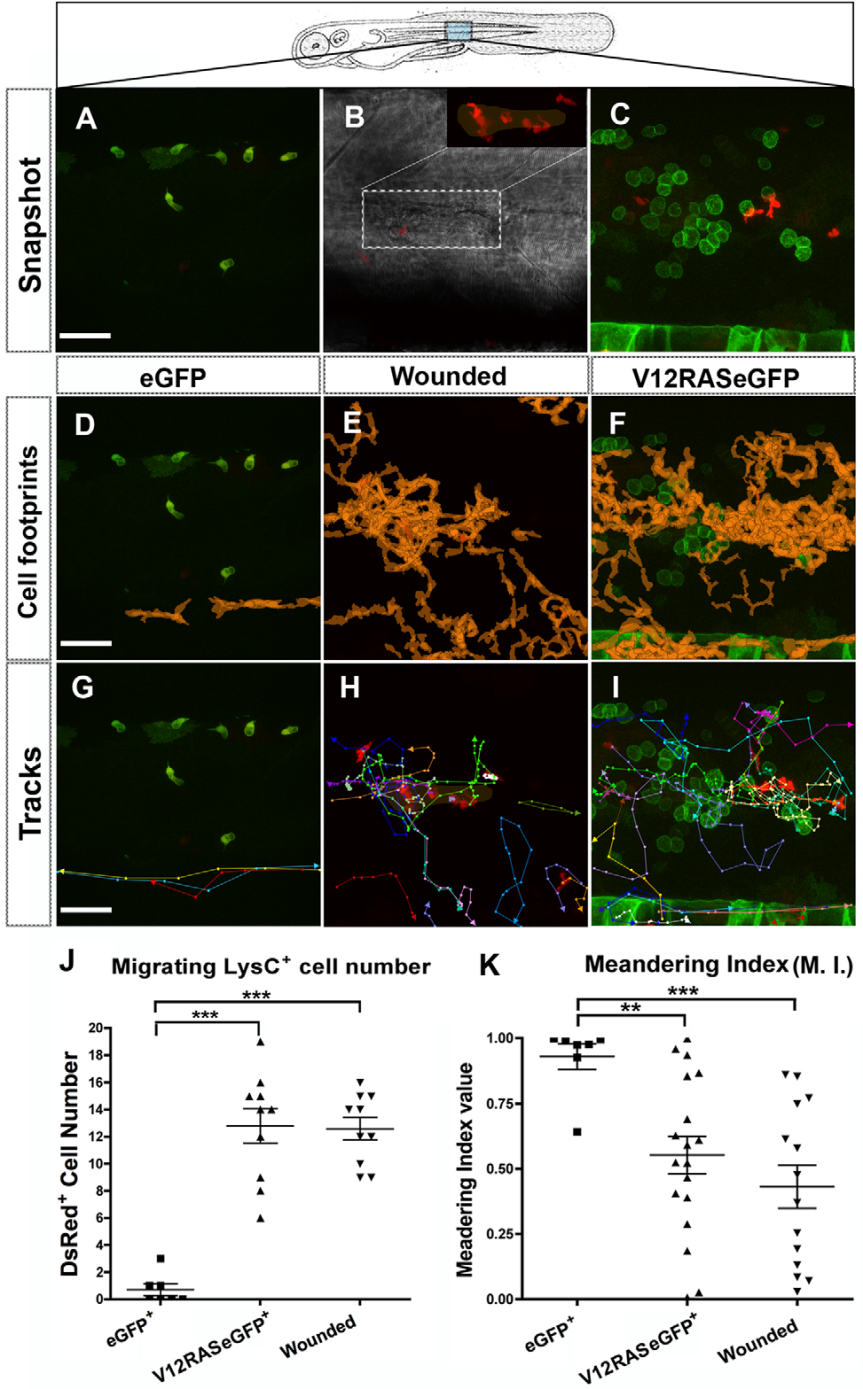
Comparison of leukocyte recruitment to V12RAS^+^ transformed cells versus to a wound. (A) Confocal image of the trunk epidermis (region indicated by schematic) from a movie of a control *Tg(kita:GalTA4, UAS:eGFP, LysC:DsRed)* larva (Video S3A). (B) DIC image of a skin laser wound in the same region of the trunk as the transformed cells in (C). Inset is a confocal image of the wound highlighting how LysC:DsRed^+^ cells are recruited to the wound center (Video S3B). (C) Equivalent image to (A) but from a *Tg(kita:GalTA4, UAS:V12RASeGFP, LysC:DsRed)* larva (Video S3C). (D–F) Superimposition of DsRed^+^ cell profiles (orange) from all time frames of 80-min time-lapse movies of (D) control, (E) wounded, and (F) V12RAS^+^ larvae, to reveal cumulative footprints (orange). (G–I) as for (D–F) but with tracks of all the LysC:DsRed^+^ cells that migrated within the field of view of the movies superimposed onto a single movie still. (J) Quantification of the numbers of LysC:DsRed^+^ cells that migrate through the imaging fields of control, V12RAS^+^, and wounded larvae. (K) As for (J) but quantification of the cell MI for each of these conditions. **, *p*<0.01; ***, *p*<0.001. Scale bars = 48 µm for all the images. In all images larvae are 4-dpf.

**Figure 3:**
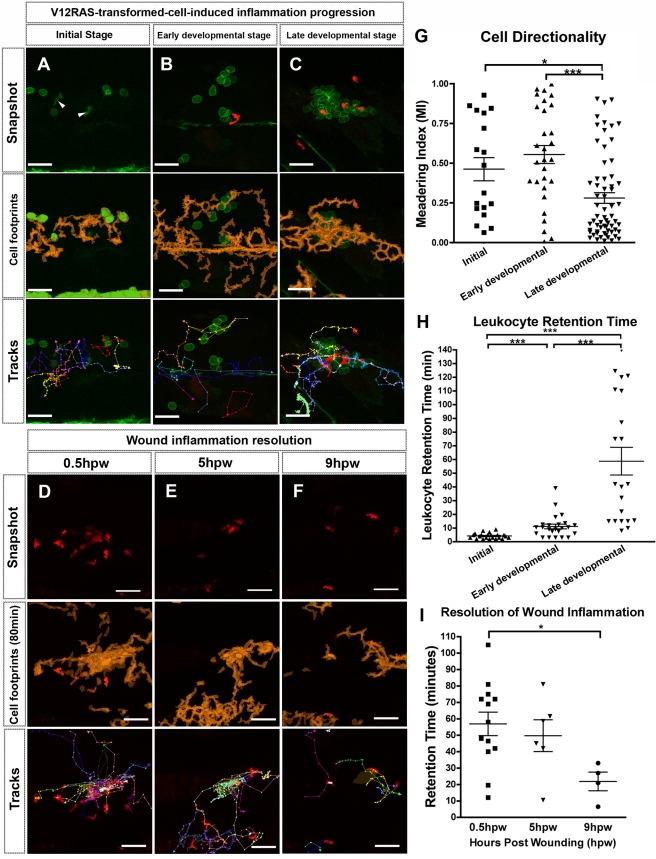
Time-lapse analysis of leukocyte response to V12RAS^+^ clone expansion versus wound inflammatory response. (A) Single image from a movie taken of the trunk region of a *Tg(kita:GalTA4, UAS:V12RASeGFP, fli:eGFP)* larva, showing an “initial stage” clone of V12RAS^+^ cells. Rounded cells with membrane-tagged GFP are V12RAS^+^ cells; solid green migrating cells are Fli:eGFP^+^ leukocytes (arrowheads) (Video S5A). (B and C) Images from “early developmental” and “late developmental” stage clones in *Tg(kita:GalTA4, UAS:V12RASeGFP, LysC:DsRed)* larvae (Video S5B and S5C). (D–F) Early (D), medium (E), and late stages (F) of flank laser wounds made to *Tg(kita:GalTA4, UAS:eGFP, LysC:DsRed)* larval skin (Video S4). Middle panels for (A–F) are the superimposed outlines of cumulated leukocyte footprints (orange) over time-lapse duration. The lower panels are superimposed tracks of all the leukocytes that have migrated through the imaging field during this period (Video S4 and S5). (G) Quantification of the MI of leukocyte migration in the presence of initial, early developmental, and late developmental stages of V12RAS^+^ clones in the epidermis. (H) Quantification of “retention time” of the leukocytes that are recruited to V12RAS^+^ clones at initial, early developmental, and late developmental stages. (I) Quantification of the “retention time” of leukocytes recruited during initial, early developmental, and late developmental stages of flank laser wounding of larval skin. *, *p*<0.05; ***, *p*<0.001. Scale bars = 48 µm.

It has not been previously reported how innate immune cells behave when they first confront transformed cells growing in the host environment. Our investigations began by comparing the response of LysC^+^ cells to V12RAS^+^ cells as they expand within normal flank epidermis, versus their response to a laser wound made in a similar region of the larval skin (Figure 2). In unwounded eGFP control 4-dpf larvae, LysC:DsRed^+^ cells migrate along the horizontal myoseptum (Figure 2A, 2D, and 2G; Video S3A) as though following a chemotactic guidance cue, with a highly polarized pathway (meandering index [MI] = 0.93±0.049, mean ± standard error of the mean). Laser wounds made to the trunk region in control larvae (without transformed cells) trigger an inflammatory response of similar intensity and duration to that previously reported for wounds made to the fin [32],[40]. An average of 12.6±0.819 (*n* = 10) LysC:DsRed^+^ cells are drawn to the wound, away from their normal patrolling route within 30 min of wounding (Figure 2B, 2E, 2H, and 2J; Video S3B). At early time points after wounding, the retention time for individual neutrophils averages about 60 min, but this decreases as the wound heals (Figure 3I). Subsequently, cells leave the wound (Figure 3D–3F; Video S4), mirroring the previously reported retrograde migration of neutrophils from wound back to the blood vessel they initially came from [32]. In equivalent stage larvae with clones of V12RAS^+^ cells growing in the epidermis, we observe many more LysC:DsRed^+^ cells in the flank region than in control larvae (Figure 2C and 2J). LysC:DsRed^+^ cells are distracted from their normal patrolling path and turn towards where the V12RAS^+^ cells are distributed (Figure 2F and 2I; Video S3C). Once in the V12RAS^+^-cell-enriched area, LysC:DsRed^+^ cells change their direction more frequently, approximating the behavior of neutrophils at a wound (Figure 2K, MI = 0.55±0.072), as a consequence of moving between individual V12RAS^+^ cell clones. They often make contact with V12RAS^+^ cells and slow down in their proximity and then migrate away again toward another V12RAS^+^ cell clone (Figure 2F and 2I; Video S3C); the total footprint of leukocytes almost entirely covers the locations of V12RAS^+^ cells (Figure 2F). The random walk behavior exhibited by LysC:DsRed^+^ cells within V12RAS^+^ cell territory, and their retrograde migration to and from individual V12RAS^+^ clones is similar to that of LysC:DsRed^+^ cells responding to a wound (Figure 2E; Video S3B). Our data indicate that transformed cell clones growing at early stages in the epidermis induce an inflammatory response and that leukocytes are recruited to the vicinity of transformed cells in a similar manner to that seen when innate immune cells sense, and are drawn towards, wounds.

We wondered whether the similar behaviors of leukocytes toward transformed cells and wounds might be reflected in molecular markers known to be associated with the changing phenotype of leukocytes at wounds [41]. In situ hybridization studies reveal that some L-plastin^+^ cells express the “alternatively activated” macrophage (M2) marker, arginase1 [42] (Figure S1C), but in similar specimens, immunohistochemistry reveals that there are also some L-plastin^+^ cells expressing “classically activated” macrophage (M1) markers such as TNFα (Figure S1D). This heterogeneity of transformed-cell-associated leukocytes is similar to the phenotypes of wound macrophage populations [41].

### Leukocytes Detect V12RAS^+^ Cells from Very Early Stages

To capture the earliest possible recognition of V12RAS^+^ cells by leukocytes we crossed *Tg(kita:GalTA4, UAS:V12RASeGFP)* fish with *Tg(fli:eGFP*) in which GFP is expressed in all early myeloid lineages [43], prior even to the onset of MPO:GFP or LysC:DsRed expression. These transgenic fish have previously been used to study how primitive macrophages are recruited to wounds [22]. Time-lapse movies revealed that the first leukocyte recruitment to V12RAS^+^ cells generally occurs as early as 55–60 hpf, when they are single cells, and no later than when they become two-cell clones (we refer to this as the “initial stage” in Figure 3A). Fli:eGFP^+^ cells migrating in the proximity of V12RAS^+^ cells appear to actively change direction, as indicated by their low MI (0.46±0.073) (Figure 3A and 3G; Video S5A), and move toward individual V12RAS^+^ cells; they occasionally pause at one clone but never for more than 10 min before moving on (Figure 3A; Video S5A). The retention time of those Fli:eGFP^+^ cells that slow down and contact V12RAS^+^ cells is short-lived, averaging 4.23±0.43 min (Figure 3A and 3H; Video S5A).

### The Expansion of V12RAS^+^ Clones Induces an Incremental Increase in Inflammatory Response That Fails to Resolve

To assess how the host inflammatory response changes as tumor expansion proceeds, we crossed V12RAS^+^/Fli:eGFP^+^ fish with *Tg(LysC:DsRed)* and chose larvae at 4–5 dpf that had already developed more than 15 V12RAS^+^ clones within the skin of one side of the flank, but with no more than 15 cells in any one individual clone. These clones we refer to as “early developmental” stage clones (Figure 3B; Video S5B). Our time-lapse movies revealed that the most significant change in inflammatory cell behavior as transformed cell clones expand is a prolonged retention time (11.15±1.69 min) at any one clone (Figure 3H). LysC^+^ cells in V12RAS^+^ clone territory at the “early developmental” stage continue to actively move between individual V12RAS^+^ clones (Figure 3B; Video S5B). The MI of these cells remains similar (MI = 0.55±0.057) to that in response to the “initial stage” V12RAS^+^ clones (Figure 3G).

Finally, we examined larvae that exhibited more than 30 V12RAS^+^ clones, with at least one clone on the flank of each fish consisting of more than 30 V12RAS^+^ cells (defined in this study as “late developmental” stage, which generally occurred when larvae were between 6 and 8 dpf), to analyze the host response to more advanced transformed cell groups (Figure 3C). At this late stage, LysC^+^ cells exhibit considerably greater retention time after contacting V12RAS^+^ cells (Figure 3H, retention time = 58.81±10.08 min; Video S5C). They are often associated with one V12RAS^+^ clone for long periods, and actively interact with individual cells within this clone (Figure 3C; Video S5C). Tracking analysis also showed a greatly reduced meandering index (Figure 3G; MI = 0.28±0.034). Our comparison of the progression of the V12RAS^+^-clone-induced immune response with that of a standard wound inflammatory response (Figure 3D–3F and 3I; Video S4) indicates that leukocytes initially respond to the V12RAS^+^ transformed cells in a way that is similar to the response to an acute wound but that this response escalates with the expansion of the V12RAS^+^ clone until it eventually progresses to a chronic inflammation state, similar to that observed in chronic wounds, or as previously described in a zebrafish model of chronic skin damage/inflammation [44],[45].

### Both Tissue Damage and V12RAS^+^ Tumor Growth Activate Multiple Leukocytic Lineages

**Figure 4:**
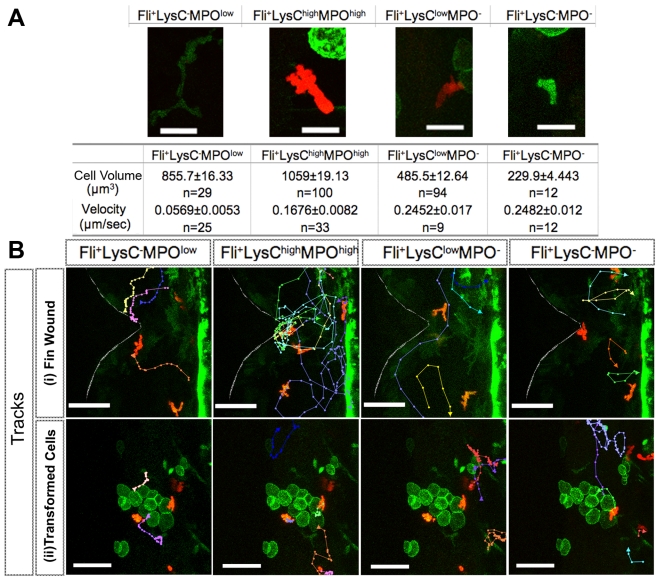
Distinguishing four leukocyte subtypes in zebrafish larvae and their response to a wound versus that to the presence of transformed cells. (A) Features of four leukocyte subtypes, as illustrated by typical examples from *Tg(fli:eGFP, LysC:DsRed)*larvae (Video S6). (B) (i) Tracks of each leukocyte subtype from a movie (Video S6A) of the wound inflammatory response in a *Tg(fli:eGFP, LysC:DsRed)* larva, illustrating how each responds to a wound. Faint white line indicates the wound edge. (ii) Similar tracking data from a movie (Video S7B) of a *Tg(kita:GalTA4, UAS:V12RASeGFP, fli:eGFP, LysC:DsRed)* larva showing that all subtypes of leukocyte are responding to V12RAS^+^ cells. Scale bars: (A), 16 µm; (B), 50 µm.

The contribution of various inflammatory cell lineages toward tumorigenesis is still a matter of considerable investigation [9],[46],[47]. In the mammal, various immune cell lineages have been found associated with the tumor microenvironment, and the composition of these leukocytes as well as their individual phenotypical changes are thought to be important in determining whether inflammation contributes to tumor elimination or tumor promotion [7],[46],[47]. We wanted to determine whether we could distinguish various innate immune cell lineages from our live imaging movies and determine how they each respond to V12RAS^+^ cells in the zebrafish larvae. By restricting our live imaging studies to within 9 dpf, we avoid the mature adaptive immune functions that could interfere with our analysis of the innate immune response [48]. We used *Tg(fli:eGFP)* to reveal all of the myeloid lineages in the early larva, and, using it in combination with LysC:DsRed, and MPO:GFP transgenes, we can distinguish four types of differently expressing leukocytes (Figure 4A; Video S6).

In agreement with studies from other groups we observed a population of leukocytes expressing MPO^low^LysC^−^, termed “tissue residential macrophages” [49] residing in the skin (Video S6A); these cells have a distinct elongated morphology and squeeze themselves between other cells. It is commonly accepted that the MPO^high^ population are neutrophils [20],[49],[50]. We found these cells also to have a high level of LysC:DsRed expression. Time-lapse movies show that neutrophils migrate with distinct polarity, often with a broad leading edge and narrow rear and that they migrate rapidly (Video S6). Amongst LysC^+^ cells there is another distinct population that are MPO^−^, which can be distinguished in time-lapse movies by their smaller size, lower level of LysC:DsRed signal, and higher migration speed compared to the MPO^high^LysC^+^ population. These cells migrate largely in a neutrophil-like manner (Figure 4A; Video S6B). Our time-lapse movies also capture another leukocyte population that are Fli:eGFP^+^ but also negative for both LysC and MPO (Figure 4A; Video S6C). These cells are smaller, less than a quarter of the volume of a neutrophil, yet they migrate with similar speed to neutrophils (Figure 4A; Video S6C), in a “neutrophil-like” manner. To examine the response of these cells to tissue damage we made a small wound in the caudal fin with a tungsten needle. Our time-lapse movies indicate that all of these various leukocytes are recruited to a wound (Figure 4Bi; Video S7A). We describe, above, how LysC^+^ cells are activated and recruited towards V12RAS^+^ cells. We next used *Tg(LysC:DsRed, fli:eGFP, kita:GalTA4, UAS:V12RASeGFP)* larvae to test whether the other subtypes of leukocytes were also activated during the transformed-cell-induced inflammatory response. Our time-lapse movies show that, just as for the wound-triggered inflammatory response, all of these leukocytes appear to be activated and drawn to V12RAS^+^ cells in the host skin (Figure 4Bii; Video S7B).

### Recruited Leukocytes Actively Interact with V12RAS^+^Cells, Initially Extending Filopodia and Later Establishing Cytoplasmic Tethers between Cells

**Figure 5:**
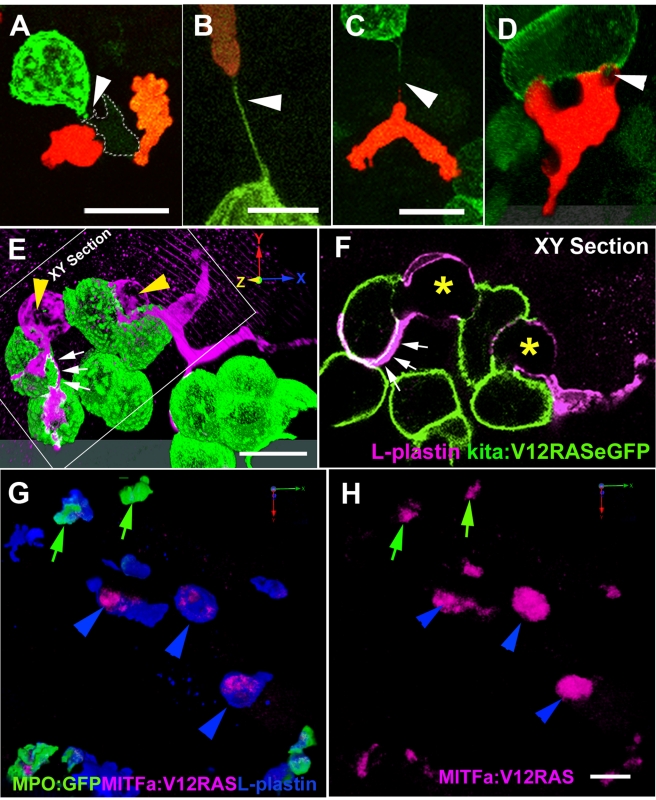
Dynamic interactions between leukocytes and V12RAS^+^ transformed cells. (A) A confocal image of a LysC:DsRed^+^ (red) cell and a macrophage (faint green with dotted outline) interacting with a V12RAS^+^ cell (green) (Video S8). (B and C) Images showing LysC:DsRed^+^ leukocytes establishing tethers with V12RAS^+^ cells, as observed in *Tg(fli:eGFP, LysC:DsRed, kita:GalTA4, UAS:V12RASeGFP)* larvae: (B) shows a tether entirely composed of V12RAS cytoplasm (green) (Video S9); (C) shows a chimeric tether composed of both V12RAS (green) and LysC:DsRed^+^ (red) cytoplasm. (D) A single image from a 3-D movie (Video S10) of a neutrophil as it glides over and engulfs pieces of a V12RAS^+^ cell; (E) 3-D reconstruction of a V12RAS^+^ cell clump (green cells) showing two macrophages (L-plastin^+^, magenta staining, yellow arrowheads) as they deform to engulf individual V12RAS^+^ cells. Small white arrows and dotted outline indicate lamellipodial protrusions extending over another pair of transformed cells. (F) A single focal plane (corresponding to that indicated in [E]) of the same pair of macrophages, showing V12RAS^+^-cell-shaped phagosomes (asterisks) within them. One macrophage has partially enveloped another V12RAS^+^ cell with a thin lamellipodial extension (indicated by small white arrows). (G) 3-D reconstruction of an anti-L-plastin-stained mitfa:V12RAS-mitfa:mCherry-injected *Tg(BACmpo:eGFP)^i114^* larval flank region, showing the presence of mCherry^+^ (red) debris within L-plastin^+^MPO^−^ cells. (H) Image as in (G) but with single channel to highlight the mCherry^+^V12RAS^+^ debris; green arrows indicate co-localization within L-plastin^+^MPO^+^ neutrophils; blue arrowheads indicate co-localization within L-plastin^+^MPO^−^ macrophages. Note that the larger clumps of debris all reside within macrophages. Scale bars = 16 µm.

We looked in more detail at the interactions that take place between V12RAS^+^ cells and recruited leukocytes. Once recruited, leukocytes actively exhibit dynamic and intimate contacts with V12RAS^+^ cells. Both transformed cells and leukocytes polarize and extend filopodial and lamellipodial protrusions towards one another (Figure 5A; Video S8). Frequently, leukocytes make extensive contact with V12RAS^+^ cells for prolonged periods of time, often more than 2 h, seemingly probing the V12RAS^+^ cells, and this behavior becomes more common as the V12RAS^+^ clones expand in number. When a leukocyte moves away from a V12RAS^+^ cell, it generally leaves a “tether” linking leukocyte and V12RAS^+^ cell; these tethers can be largely transformed-cell-derived, or occasionally have equal contributions from immune cell and transformed cell (Figure 5B and 5C; Video S9). Occasionally, a leukocyte appears to be dragged back to the V12RAS^+^ cell by this tether between the two cells (Video S9).

### Neutrophils and Macrophages Show Distinctive Engulfment Behaviors

Activated innate immune cells are known to engulf both cell debris and invading pathogens at sites of tissue damage and infection [23]. In our time-lapse movies we observe active phagocytosis by all of the LysC^+^ cells and tissue residential macrophages in the presence of V12RAS^+^ clones. Perhaps unsurprisingly, the different leukocyte lineages engulf in different ways. LysC^+^ cells (neutrophils) appear to extend membrane protrusions and break off small fragments of V12RAS^+^ cells (Figure 5D; Video S10), whereas macrophages are able to deform themselves to engulf a whole cell (Figure 5E and 5F). The consequences of these two phagocytic modes is most clearly indicated by L-plastin staining of tissues, which reveals that cells labeled by L-plastin, but not MPO:GFP (i.e. macrophages), contain larger aggregates of engulfed RFP-tagged (V12RAS^+^) material resembling intact cells, while MPO^+^ cells (neutrophils) contain only smaller fragments of engulfed V12RAS^+^ cells (Figure 5G and 5H). To test whether immune cells were not simply engulfing transformed cells because the latter were undergoing apoptosis, we stained for the cleaved (active) form of caspase 3 and observed no indication of increased apoptotic cells within these early transformed cell populations or the immune cells drawn to them (data not shown).

### V12RAS^+^ Cells Trigger an Increase in H_2_O_2_ Production, Which Leads to Leukocyte Recruitment

**Figure 6:**
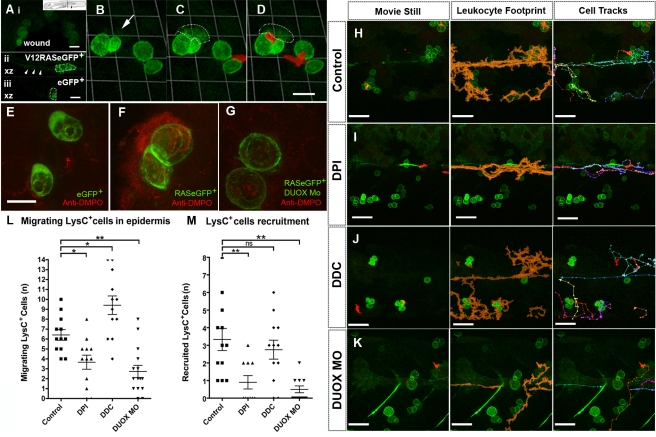
Local synthesis of H_2_O_2_ is required for leukocyte recruitment toward V12RAS^+^ cells. (A) (i) Wound edge of a larva pre-loaded with H_2_O_2_-indicating dye (acetyl-pentafluorobenzene sulphonyl fluorescein) to reveal H_2_O_2_ synthesis (green), 10 min after wounding; the wound location corresponds to the arrow in inset schematic; (ii) and (iii) Confocal *Z*-sections (corresponding to the transect line in inset) through the skin of larvae pre-loaded with acetyl-pentafluorobenzene sulphonyl fluorescein. (ii) is from a V12RAS^+^ larva, with dotted outline indicating the V12RAS^+^ cells. Arrowheads indicate increased H_2_O_2_ signal in the skin of the V12RAS larva. (iii) is a control larva with non-transformed eGFP-expressing mucus-secreting cells outlined, indicating reduced background H_2_O_2_ levels. (B–D) Stills from a time-lapse movie showing transient H_2_O_2_ production and subsequent recruitment of LysC:DsRed^+^ cells towards V12RAS^+^ cells. Arrow in (B) and dotted line in (C) and (D) indicate the region where H_2_O_2_ pulse occurs prior to LysC:DsRed^+^ cell recruitment (Video S11). (E–G) Anti-DMPO antibody (red “mist”) detection of DMPO-protein adducts in larvae with control eGFP-expressing goblet cells (E), V12RASeGFP-transformed goblet cells (F), and V12RASeGFP-transformed goblet cells with DUOX morpholino knockdown (G). (H–K) Analysis of time-lapse movies from control (H), DPI-treated (I), DDC-treated (J), and DUOX-morpholino-injected (K) V12RAS^+^LysC^+^ larvae. In (H–K) the left-hand panel shows a still image from the time-lapse movie (Video S12), the middle panel shows the superimposed, cumulative footprints (orange) of all the leukocytes during the 120-min period of the movie, and the right-hand panel shows the superimposed tracks of all the leukocytes that migrated through the imaging field during this period. (L) Quantification of LysC:DsRed^+^ cell number during the 120-min time course of the movie. (M) Quantification of LysC:DsRed^+^ cell contacts with V12RAS^+^ cells during the same 120-min time course. *, *p*<0.05, **, *p*<0.01; ns, not significant. Scale bars: (A–D), 24 µm; (E–G), 16 µm; (H–K), 50 µm.

Our time-lapse observations suggest that early recruitment of innate immune cells towards V12RAS-transformed cells in the larval epidermis may be similar to the wound inflammatory response. We next wanted to know how V12RAS^+^ transformed cells might attract the attention of host innate immune cells, and whether there were parallels between the two types of inflammatory triggers—wounding and the presence of transformed cells. A recent study showed that H_2_O_2_ is released after wounding of zebrafish larvae and that the consequent gradient of H_2_O_2_ is required for leukocyte recruitment towards the wound [26]. We wanted to test whether V12RAS^+^ cell growth in the epidermis might utilize the same signal to activate immune cells. We soaked larvae in the H_2_O_2_-specific probe acetyl-pentafluorobenzene sulphonyl fluorescein (which is converted to its fluorescent form when exposed to H_2_O_2_) [51] for 30 min prior to imaging. The efficiency of this reporter dye was confirmed by imaging the transiently increased levels of H_2_O_2_ at wound edges of control larvae (Figure 6Ai; [26]). We observed slightly increased levels of fluorescent signal throughout V12RAS^+^ larval flank skin as compared with control larvae (Figure 6Aii and 6Aiii). But more dramatically, our time-lapse studies revealed stochastic and transient H_2_O_2_ production in the vicinity of V12RAS^+^ cells and their immediately neighboring normal cells (Figure 6B–6D; Video S11), and this transient increase of H_2_O_2_ signal generally appears to precede the recruitment of several LysC:DsRed^+^ cells toward the V12RAS^+^ clone (Figure 6B–6D; Video S11). These data indicate that H_2_O_2_ could play a role in V12RAS^+^-induced leukocyte recruitment. To complement this real time approach to imaging H_2_O_2_ flux in the neighborhood of transformed cells, we also utilized an immuno-spin trap technique that reports a history of ROS exposure by accumulation of 5,5-dimethyl-l-pyrroline N-oxide (DMPO) protein adduct wherever protein-centered radicals are generated [52]; incubation of larvae in medium containing DMPO for 6 h and subsequent immunostaining for DMPO indicates a “mist” of ROS modification around all V12RAS^+^ cells but none adjacent to control eGFP cells (Figure 6E and 6F).

To test whether H_2_O_2_ is indeed required for leukocyte recruitment to transformed cells, we used a pan-NADPH oxidase inhibitor, diphenyleneiodonium chloride (DPI), which has previously been shown to block H_2_O_2_ production in wounded zebrafish larvae, and thus prevent leukocyte wound recruitment [26]. At the same concentration that blocks leukocyte migration toward a wound, we see reduced numbers of LysC:DsRed^+^ cells migrating into the epidermis of DPI-treated V12RAS^+^ larvae compared with control larvae (compare Figure 6H and 6I; quantification in Figure 6L; Video S12A and S12B); LysC:DsRed^+^ cells that migrate along the horizontal myoseptum no longer change their direction to migrate towards the V12RAS^+^ cells, as they do in untreated larvae (compare Figure 6E and 6F; quantification in Figure 6J; Video S12A and S12B).

H_2_O_2_ can be generated either directly by specialist NADPH oxidases (NOXes) or converted by superoxide dismutase (SOD) from superoxide (O_2_^−^) that has been generated by other NOXes [53]. Treating 4-dpf V12RAS^+^ larvae with the SOD inhibitor diethyldithiocarbamate (DDC) [54] will drive increased superoxide anion (O_2_^−^) levels and reduce any H_2_O_2_ generated via SOD [53], and while we see increased numbers of neutrophils in the skin after this treatment (Figure 6H, 6J, and 6L), we see no change in their recruitment to V12RAS^+^ clones (Figure 6H, 6J, and 6M; Video S12A and S12C). These data suggest that SOD conversion of O_2_^−^ to H_2_O_2_ is not required for transformed cell recruitment of leukocytes.

In zebrafish larvae, the sole NOX that can generate H_2_O_2_ independently of SOD is DUOX, and morpholino knockdown of DUOX has been shown to prevent recruitment of neutrophils to wounds [26]. We used morpholinos to knock down zebrafish DUOX by blocking pre-mRNA splicing and showed by RT-PCR that this knockdown extended at least until 5 dpf (Figure S2). Immuno-spin trap analysis of such morphant embryos revealed a much reduced “mist” of ROS modified proteins adjacent to V12RAS^+^ cells (Figure 6G), indicating H_2_O_2_ generation had successfully been blocked. Analysis showed that leukocyte recruitment to V12RAS^+^ clones in DUOX morphant larvae appeared similar to that in DPI-treated larvae, with a much reduced number of LysC:DsRed^+^ cells seen migrating within the epidermis and very few of these cells passing close to V12RAS^+^ cells (Figure 6K; Video S12D). For both DPI and DUOX morpholino treatments, leukocytes otherwise exhibit entirely normal motility; for example, their migration along the horizontal myoseptum is unperturbed. Together these data indicate that where V12RAS^+^ cells are growing in the host epidermis, increased H_2_O_2_ production is the primary signal drawing leukocytes to the locale of these transformed cells, just as is the case for recruitment of inflammatory cells to wounds. But these experiments alone do not reveal whether it is the transformed cells themselves or their immediate disturbed neighbors that generate the H_2_O_2_ signal.

### Both Transformed Cells and Their Neighbors Generate the H_2_O_2_ Attractant

**Figure 7:**
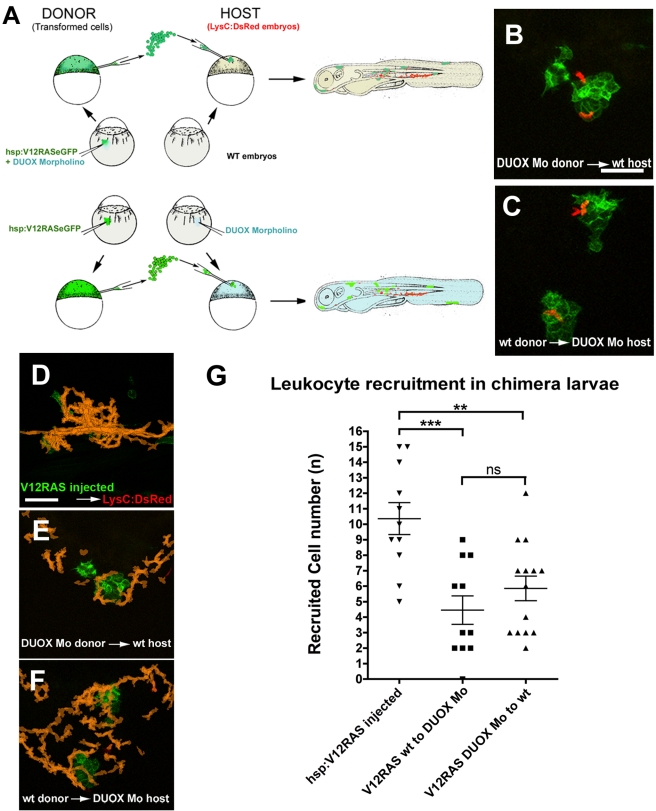
Analysis of leukocyte recruitment in chimeric V12RAS-transformed cell-bearing larvae. (A) Schematic diagram to illustrate how we generate chimeric V12RAS^+^ transformed cell-bearing *Tg(LysC:DsRed)* larvae, in which either the donor transformed cells or host tissues are morphant for DUOX. (B) A still image from Video S13B illustrating how LysC:DsRed*^+^* cells are recruited to DUOX morphant V12RAS^+^ cells in WT host environment. (C) A still image from Video S13C showing LysC:DsRed*^+^* cells recruited to WT V12RAS^+^ cells in a DUOX morphant host environment. (D–F) Cumulative LysC:DsRed*^+^* cell footprints (orange) of representative movies shown in Video S13. (G) Graph to illustrate numbers of recruited LysC^+^ cells drawn towards V12RAS^+^ transformed cells in the two transplantation scenarios above compared with pTol2-hsp:V12RASeGFP injected into *Tg(LysC:DsRed)* control. **, *p*<0.01; ***, *p*<0.001; ns, not significant. Scale bars = 50 µm.

To determine the cellular source of the H_2_O_2_ signal, we undertook a series of transplantation experiments to generate chimeric DUOX morphant/wild-type [WT] larvae (Figure 7). In a pair of complementary experiments we transplanted cells from embryos previously injected with hsp:V12RASeGFP into embryos expressing LysC:DsRed, where either the donor or the host embryo had been previously injected with DUOX morpholinos. In this way we generated larvae with green transformed cells and red neutrophils in which either the transformed cells or their neighbors were deficient in generating H_2_O_2_ via DUOX (Figure 7B and 7C; Video S13B and S13C). We found that, in both cases, neutrophils were drawn to the vicinity of transformed cells in numbers that were not significantly different from one another (although less than for positive control hsp:V12RASeGFP-injected larvae—Figure 7D–7G; Video S13), suggesting that both V12RAS over-expressing cells and neighboring host epithelial cells use DUOX to generate H_2_O_2_ that recruits leukocytes. LysC:DsRed embryos with transplanted control GAP43-eGFP-expressing cells showed no sign of immune cell recruitment to eGFP^+^ cells (Video S14). We observed, however, that in those chimeric embryos where transformed cells were deficient in DUOX, leukocytes, whilst drawn towards the clone, tended to skirt around the transformed cells, rather than over them as in the converse or positive control experiments (Figure 7D–7F; Video S13).

### Blocking the Host Inflammatory Response to Oncogene-Transformed Cells Leads to Reduced Transformed Cell Numbers

**Figure 8:**
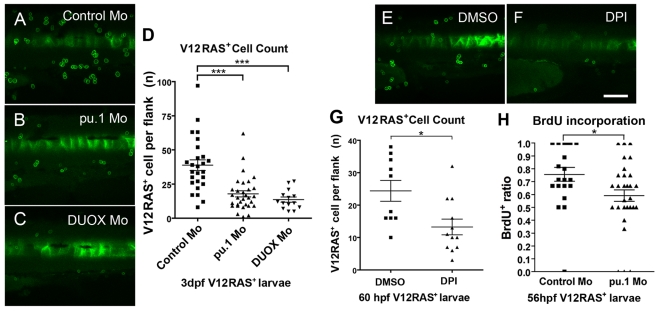
Analysis of transformed cell clones in larvae where immune cell interaction is disrupted. (A–C) Typical confocal *Z*-projections of 3-dpf V12RAS^+^ larvae that were previously injected with (A) control (*n* = 26), (B) pu.1 (*n* = 31), and (C) DUOX (*n* = 14) morpholinos. (D) Quantification of the numbers of V12RAS^+^ cells in equivalent flank skin within these three groups at 3 dpf. (E and F) Typical confocal *Z*-projection images of control and DPI-treated larvae at 60 hpf for similar analysis of V12RAS^+^ cells. (G) Quantification of V12RAS^+^ cell numbers in control (*n* = 10) versus DPI-treated (*n* = 12) larvae at 60 hpf; *, *p*<0.05; ***, *p*<0.001. (H) Graphic representation of BrdU incorporation by V12RAS^+^ cells in pu.1 morphant (*n* = 33) versus control (*n* = 21) larvae. Each point on the scatter plots represents one larva. Scale bar = 150 µm.

Having revealed that innate immune cells are actively recruited from very early stages to sites of oncogene-transformed cells, and that they extensively interact with them, we sought to test the outcome of these interactions by preventing contact between these cells. We have done this in three complementary ways. First, we used morpholino knockdown of pu.1, as previously described [55], to transiently block the differentiation of myeloid lineage cells; in such larvae, myeloid cells are delayed from appearance until 3 dpf ([55] and our own observations). This approach directly tests the requirement of innate immune cells for growth of transformed cell clones. Second, we used a chronic exposure to the pan-NOX inhibitor DPI (36–60 hpf). And, third, we again generated DUOX morphants to specifically block the attractant signal. The latter two strategies carry the proviso that they may also influence V12RAS^+^ cell proliferation directly because of potential ROS requirements for RAS-transformed cell growth [56],[57]. In all three of these treatments that prevent recruitment of immune cells to the transformed cells, we see a consequent reduction in numbers of V12RAS^+^ cells (Figure 8A–8G), suggesting a significant trophic role for host innate immune cells during the earliest stages of transformed cell development. To test whether this reduction in numbers of transformed cells in the absence of immune cell recruitment is a consequence of increased apoptosis or reduced proliferative index, or both, we have immunostained for the cleaved (active) form of caspase 3, which indicates almost no apoptosis in either pu.1 morphant or control transformed cells over this period (data not shown), but we do see a significant reduction in the proliferative index of transformed cells, as revealed by 6-h BrdU incorporation rate, from 76% in control V12RAS^+^ larvae (*n* = 21) to 59% in pu.1 V12RAS^+^ morphants (*n* = 33) (Figure 8H), suggesting that the immune cell trophic signal operates by enhancing cell proliferation.

## Discussion

Cancer progression begins when a transformed cell overgrows within otherwise healthy normal tissue; this cell must then somehow escape host immunosurveillance and subsequently hijack the neighboring tissue architecture to parasitize the host. Finally, it may seed off metastatic offspring that will invade other sites of the body [2]. All of these steps involve intimate interactions with the host tissues, and several steps will inevitably involve interactions with, or avoidance of, the host innate immune system, which functions both as a potential “surveillance” mechanism via immune elimination but also as a source of cells that might be subverted to suppress anti-tumor immunity and aid tumor progression and spread [3],[7],[8],[58]. In mammals there is good evidence suggesting that several subtypes of bone-marrow-derived myeloid lineages have a direct role in promoting the establishment of a microenvironment that enables, or even encourages, tumor growth [47],[59],[60].

Multiphoton imaging studies in mouse tumor models have revealed that tumor-associated macrophages directly interact with cells of later stage tumors and can function as “pilots” to guide individual tumor cells away from the primary cancer to nearby vessels [9]. More recently, in a murine lung metastasis tumor model, ex vivo live imaging of intact lungs revealed that macrophages are required for efficient tumor cell extravasation at the site of metastasis exit [61]. However, in mouse it is much less easy to investigate the earliest host cellular response toward genetically aberrant cells as the cells first emerge in otherwise normal tissue.

In the current study, we have taken advantage of the translucency of zebrafish larvae to reveal the dynamics of these very early interactions (that we describe as developmental stages) between host innate immune cells and the early transformed precursors of tumors. Since the first T cells are not born until 2 wk post-fertilization in zebrafish, and mature T cells appear even later [24], we are studying a time window in zebrafish larval development when the function of innate immune cells can be observed without interference from adaptive immunity, which, at later stages, will almost certainly play further roles in cancer progression.

### Transformed Cells Are Rapidly Sensed by Innate Immune Cells

What is immediately clear from our studies is the very early onset of an innate immune response toward transformed cells, almost as soon as the oncogenic transformation has commenced in otherwise normal tissue. Two distinct transformed lineages (melanocytes and goblet cells) are seen to recruit both neutrophils and macrophages (and potentially other leukocytic lineages) at stages as early as when clones consist of only singletons or doublets. These data indicate that oncogene-transformed cells, even as individuals, can emit cues that, directly or indirectly, trigger a host innate immune cell inflammatory response.

Many clinical studies show that pro-inflammatory factors are elevated in the serum of cancer patients [62]. Studies in mouse models have also shown that oncogene-induced release of pro-inflammatory cytokines can act as a paracrine signal that might promote an inflammatory microenvironment in the surrounding tumor stroma, which in turn drives tumor growth [62],[63]. We have also found in our V12RAS-driven zebrafish melanoma model that many pro-inflammatory factors are up-regulated in established tumor tissues (P. Walker, M. Jones, S. He, C. Michailidou, N. Haud, et al., unpublished data). Importantly, we also see up-regulation of pro-inflammatory factors in the early stage larvae, even before establishment of a mature tumor, suggesting that pro-inflammatory factors may play some role in regulating the initial phase of an oncogene-driven inflammatory response by paracrine mechanisms.

### A Central Role for H_2_O_2_ as a Regulator of Inflammatory Responses to Epithelial Tissue Insults

An immediate question that arises from our in vivo observation concerns the nature of the signal(s) that direct leukocyte recruitment. Our data suggest that the key signal regulating leukocyte activation and recruitment towards transformed cells is DUOX-mediated H_2_O_2_ production, which is also the immediate damage signal that attracts neutrophils to wounds [26]. Studies in mammalian airway epithelium also suggest a regulatory role for enhanced H_2_O_2_ production in the host innate immune response to diverse bacterial triggers [64]. Thus, enhanced production of H_2_O_2_ might be a universal mechanism used by the host to sense the disruption of tissue homeostasis by various insults and to activate the appropriate innate immune response. The current data indicate that in this instance, DUOX is the major contributor to the synthesis of H_2_O_2_ at sites where V12RAS-transformed cells are dividing but clearly other NOXes may also contribute and, indeed, may play more significant roles where other cell lineages or transforming oncogenes are involved.

Our transplantation experiments show that both oncogene-transformed cells and their disrupted neighbors contribute to the signal that attracts immune cells to these sites. It has previously been shown that oncogenic RAS-transformed cells suffer ROS stress [65], but what is not clear is how individual malignant cells disrupt tissue homeostasis in order to trigger otherwise healthy, epithelial neighbors to do likewise. Perhaps, the upstream signal that directs DUOX activation in these cells is the same as that which triggers H_2_O_2_ synthesis by wound edge epithelial cells and will turn out to be a generic, rapid response to tissue disruption. In a wound scenario, any NOXes must already be expressed so that tissue damage merely triggers their activation. However, there is evidence that colon cancer cells up-regulate NOX1 [66], and so it is possible that DUOX is up-regulated by transformed cells in our fish model also, although our qPCR data would suggest that this is not the case (data not shown), and recent studies indicate that DUOX is constitutively expressed in zebrafish larval mucosal epithelia, including epidermis [67]. Also entirely unclear is how leukocytes might read the chemotactic H_2_O_2_ signal or some downstream consequence of it. There is no known receptor for H_2_O_2_, but potentially H_2_O_2_ gradients might be “read” via intracellular sensors such as phosphatases that subsequently regulate the chemotactic response. Indeed, one such phosphatase, PTEN, which is known to have a pivotal role in cell migration, is modulated by exposure to H_2_O_2_ [68], and other phosphatases clearly have an impact on growth factor signaling and might alter the chemotactic response in this way also [69]. Alternatively, there may be other extracellular (or intracellular) substrates that H_2_O_2_ modifies in some way to convert them into chemotactic factors or other modulators of cell motility [53],[70], and the genetic tractability of zebrafish offers good opportunities to uncover such mechanisms.

### Unpicking the Roles of Innate Immune Cells in the Early Steps of Tumor Development

Aside from a shared molecular mechanism that mobilizes host innate immune cells towards these “early tumors” and to wounds, our live imaging observations offer further support for, and refinement of, the well-established doctrine suggesting that “tumors are wounds that do not heal” [13],[71]. While the earliest recruitment of leukocytes resembles that of an acute healing wound, as transformed clone size increases, so the time spent by leukocytes with the clone also increases until their behavior more resembles that of a chronic skin lesion where inflammation does not resolve [44],[45]. This prolonged time spent in the neighborhood of transformed cells may lead—as well as to the changes in dynamic cell behavior we describe here—to phenotypic changes in leukocytes. Indeed, we see some leukocytes expressing the M2 marker, arginase1, which indicates a more trophic function for those cells at the transformed cell site. The molecular nature of such trophic signals is not revealed by our studies, but clearly such transitions may significantly influence tumor progression, and the translucent zebrafish offers potential opportunities to live image these events in situ, using appropriate reporter fish lines.

It has been shown in mouse models that tumor-recruited bone-marrow-derived cells are a heterogeneous population composed of many subtypes of leukocytes distinguished by their surface markers, but there is no definitive association between the presence of any individual subtypes and a particular outcome of tumor progression [47]. Definitive markers for cataloging the zebrafish larval myeloid cell subtypes are still subject to refinement. However, by combining several transgenic reporter fish lines for different myeloid lineage marker genes and cell morphology, our observations reveal four different subtypes of leukocyte that show a range of behaviors during their recruitment to, and interaction with, V12RAS^+^ cells, and suggest that multiple leukocyte lineages can participate in the earliest inflammatory response to transformed cells. Our data reveal transformed cell material in both neutrophils and macrophages, but macrophages appear by far the more significant phagocytes of these two leukocyte lineages. The high-resolution live imaging also enables us to observe the structures that transiently form between innate immune cells and transformed cells. For example, we see tethers linking leukocytes and transformed cells, and we speculate that these may play a “holding” role, enabling leukocytes to maintain a long-term association with transformed cells. Similar tethers, termed nanotubes, have been previously reported to play important roles in immune cell-cell communication in vitro [72], and the same may be true for these links between immune cells and transformed cells in vivo.

For the first time, to our knowledge, we have been able to observe the behaviors of various innate immune cell lineages as they interact with these developing transformed cell clones, and, using several complementary strategies, we go on to demonstrate that these interactions have significant impact, because without them, we see reduced proliferation and, as a consequence, fewer transformed cells, at least in the early stages we have examined. This demonstration of a trophic role is all the more dramatic because of our observation that when recruited to a site where transformed cells are growing, neutrophils and macrophages are able to phagocytose some of these cells, so there appear to be competing clearance and trophic roles for innate immune cells at these earliest stages of tumor initiation. However, at least, in simple terms of the numbers of surviving transformed cells, we show that the presence of innate immune cells at the lesion site is beneficial to early transformed cell growth. Our current function testing studies are acute experiments, but other approaches may enable longer term blocking of recruitment of innate immune cells, or more permanent genetic deletion of various leukocyte lineages, which will allow us to dissect precisely the contribution of each lineage to tumor initiation, clearance, and trophic support, from these early initiation stages all the way through to full-blown cancer.

The in vivo, live observations extend our current understanding of the earliest surveillance steps whereby innate immune cells sense the presence of small numbers of oncogene-transformed cells in otherwise normal tissue. Besides providing opportunities to live image these early interactions, our zebrafish model also enables us to study the signals that regulate leukocyte recruitment to these oncogene-transformed cell precursors of tumors, and we reveal that H_2_O_2_ is a convergent signal for initiating the host inflammatory response to both oncogene-transformed cells and to wounds, thus further extending the parallels between cancers and healing wounds.

## Materials and Methods

### Zebrafish Strains and Maintenance

Adult zebrafish (*Danio rerio*) were maintained and crossed as previously described [73]. Strains included Lon AB (obtained from Robert Kelsh, University of Bath); *Tg(UAS:eGFP-H-RASV12)io6* [38], *Et(kita:GalTA4,UAS:mCherry)hzm1* [37], *Tg(fli:eGFP)* [43], *Tg(LysC:DsRed)* [33], and *Tg(BACmpo:eGFP)^i114^* [31], and embryos were maintained and staged according to standard protocols [73].

### Wounding Assays

Sterile laser wounding was performed as previously described [22] with adaptation for 5-dpf larvae. In brief, zebrafish embryos at 5 dpf were anesthetized in 0.3% Danieau's solution containing 0.1 mg/ml Tricaine (ethyl 3-aminobenzoate, Sigma). Instead of targeting the laser beam to the yolk, wounds were made to flank skin within the area illustrated in Figure 2. Fin wounds were made as previously described [40], using tungsten needles (Fine Science Tools) on 4-dpf embryos.

### Oncogene-Induced Cell Transformation by DNA Injection

For our transient V12RAS- and v-Src-induced cell transformation experiments, embryo injections were performed according to the published protocol [29],[34]. In brief, for RAS transformation, LysC:DsRed embryos were co-injected with 1 nl of plasmid containing mitfa:V12RAS-mitfa:mCherry (25 ng/µl) and 10 U I-SceI meganuclease at one-cell-stage. Embryos containing mCherry^+^ melanocytes clones were selected at 48 hpf for further live imaging analysis. For v-Src-induced cell transformation pBR-Tol2-UAS-GAP43-eGFP-SC-v-Src, pCS2-Gal4FF (25 ng/µl) and Tol2 capped RNA (100 ng/µl) were co-injected into one-cell-stage LysC:DsRed embryos at 2 nl per embryo. Equivalent amounts of pBR-Tol2-UAS-GAP43-GFP, pCS2-Gal4FF and Tol2 capped RNA mix were used for control injections. After injection, embryos developed in normal conditions, and at 3 dpf embryos with similar GFP^+^ cell clones were selected for live imaging.

### Morpholino Injection

All the morpholinos were obtained from GeneTools. Splicing block morpholino against zebrafish DUOX (5′-AGTGAATTAGAGAAATGCACCTTTT-3′), 0.4 mM, or standard control morpholino, 0.4 mM, was injected together with 0.2 mM p53 morpholino (5′-GCGCCATTGCTTTGCAAGAATTG-3′) into one-cell-stage embryos, as previously described [26]. We injected morpholino against zebrafish pu.1 (5′-GATATACTGATACTCCATTGGTGGT-3′) as previously described [55].

### Morphotyping, RT-PCR, and qPCR

For all RNA preparations we used phenol-chloroform extraction (TRIzol, Invitrogen), and cDNA template was generated using SuperScript VILO cDNA synthesis kit (Invitrogen). For morphotyping of embryos/larvae, RNA was prepared from 3-dpf, 5-dpf, control and DUOX morphant larvae. Knockdown efficiency was confirmed using the following primers: DUOX forward, 5′-ACACATGTGACTTCATATCCAG-3′, and DUOX reverse, 5′-ATTATTAACTCATCCACATCCAG-3′. DUOX-morpholino-mediated splice perturbation produced a 39-bp in-frame deletion within the peroxidase-like domain of DUOX as described before (Figure S2) [26].

For RT-PCR of pro-inflammation genes, total RNA was prepared from pools of five each of RAS^+^ larvae and their RAS^−^ siblings at 5dpf. The primers used were as described previously [74]: *il8*F 5′-TGTGTTATTGTTTTCCTGGCATTTC-3′, *il8*R 5′-GCGACAGCGTGGATCTACAG-3′; *cxcl1*F 5′-GGCATTCACACCCAAAGCG-3′, *cxcl1*R 5′-GCGAGCACGATTCACGAGAG-3′; *ifn1*F 5′-TCTTAATACACGCAAAGATGAGAACT-3′, *ifn1*R 5′-GTCAGGACTAAAAACTTCAC-3′; *tnf*αF 5′-GCGCTTTTCTGAATCCTACG-3′, *tnf*αR 5′-TGCCCAGTCTGTCTCCTTCT-3′; *il1*βF 5′-GCCTGTGTGTTTGGGAATCT-3′, *il1*β R 5′-TGATAAACCAACCGGGACAT-3′; *zfef*αF 5′-CTGGTTCAAGGGATGGAAGA-3′, *zfef*αR 5′-GAGACTCGTGGTGCATCTCA-3′.

For qPCR of V12RAS-associated pro-inflammation genes we heat shocked (39°C for 30 min) 5-dpf WT and *Tg(hsp70l:eGFP-HRAS_G12V)io3* larvae. After 6 h, total RNA was extracted from pools of 60 larvae, and qPCR was performed with LightCycler 480 II thermocycler (Roche) using the SYBR Green I Master kit (Roche). The conditions for the reactions were as follows: 10 min at 95°C, followed by 40 cycles of 15 s at 95°C and 60 s at 60°C. For each sample, expression levels were analyzed using Q-Gene software, which expresses data as mean normalized expression. All the genes were normalized to expression of β-actin as an endogenous control. The plot is a mean of three different samples.

### Generating Chimeric DUOX Morphant/WT Embryos by Transplantation

Blastomere transplantation experiments were performed according to standard procedure [73]. In brief, embryos were injected at the one cell stage with pTol2-hsp:V12RASeGFP (25 ng/ul)+Tol2 capped RNA (100 ng/ul) with and without DUOX morpholino+p53 morpholino, and used as donors. Negative control donors were prepared by injecting pBR-Tol2-UAS-GAP43-eGFP+Gal4FF capped RNA+Tol2 capped RNA. Approximately 50 donor cells were transplanted into stage-matched WT LysC:DsRed host embryos or DUOX-morpholino-injected LysC:DsRed host embryos (Figure 7A). Transplantations were carried out between 4 hpf and 5 hpf. After transplantation, healthy chimeric embryos were left to develop in Danieau's solution containing antibiotic Pen/Step in the 28.5°C incubator until 20 hpf, when all the chimeric embryos were heat shocked at 37°C for 1 h to induce expression of the V12RAS transgene. The chimeric larvae were repeatedly heat shocked for 1 h every 8 h. At 3 dpf, larvae were selected that had clones of superficial GFP-expressing cells for live imaging studies (as described below). To generate positive mosaic control larvae, we injected 1 nl of pTol2-hsp:V12RASeGFP (25 ng/ul)+Tol2 capped RNA (100 ng/ul) into a single cell of stage 16- to 32-cell *Tg*(*LysC:DsRed*) embryos.

### Whole Mount In Situ Hybridization and Whole Mount Immunofluorescence

Whole mount fluorescent in situ hybridization was performed as described before [75], with some modification. To generate a zebrafish *arginase1* probe we first cloned the gene from pooled zebrafish cDNA into pCRII-TOPO (Invitrogen), using the primers Arg1F 5′-ATGATGAAGATGAAGAGCCTTAGCG-3′, Arg1R: 5′-TTATGGATTTGGCATTTTGTAATCTGGG-3′. The pCRII-zfArg1 construct was linearized using BamHI, and T7 polymerase was used to generate DIG-labeled (Roche) probe. V12RAS^+^ larvae, 5 dpf, and their RAS^−^ siblings were used for in situ analysis. Hybridization and post-hybridization washes of 5-dpf larvae were carried out at 68°C and the signal developed using a Fluorescein-Tyramide TSAplus kit (PerkinElmer) according to the user manual, with some modification according to published protocol [75]. After the in situ signal had developed, samples were re-fixed in 4% PFA for 30 min then washed in PBST for 3× 10 min before carrying out subsequent immunostaining to reveal leukocytes and RAS^+^ cells.

Immunostaining for L-plastin was performed as previously described [40]; in brief, embryos/larvae were fixed in 4% PFA plus 0.2% Triton X-100 at room temperature for 2 h prior to rinsing, blocking, and incubation with rabbit polyclonal anti-L-plastin antibody (1∶500) overnight at 4°C. Other primary antibodies used in this study were mouse anti-TNFα (1∶10) (ab1793, Abcam) and mouse anti-RAS (1∶100) (#610001, BD Transduction Laboratories). Subsequently, either a Cy3- or Cy5-conjugated secondary antibody (Jackson Laboratories) was used to reveal primary antibody localization. For analysis of leukocyte recruitment, embryos were segregated into RAS^+^ and RAS^−^ groups, and L-plastin^+^ cells in the same flank skin region were counted for each embryo using a Zeiss Axiophot microscope.

### Cell Proliferation Analysis by BrdU Incorporation

For BrdU labeling, we incubated 50-hpf larvae in 10 mM BrdU in E3 fish water for 6 h. Following fixation in 4% PFA, larvae were treated with 2 N HCl for 20 min, before they were processed for immunofluorescence with anti-BrdU antibody (Sigma). Cy3- or Cy5-conjugated secondary antibody was used to reveal BrdU uptake in GFP^+^ (i.e., V12RASeGFP or control eGFP-expressing) cells in either pu.1 morphant or control larvae.

### Live Imaging and Time-Lapse Imaging of Zebrafish Embryos

For all of our live imaging studies, larvae were mounted on their sides in 1.5% low melting agarose (Sigma), in a glass-bottomed dish, filled with 0.3% Danieau's solution containing 0.01 mg/ml Tricaine. The climate chamber covering the microscope stage was set at 28°C. Images were collected using a Leica SP5-AOBS confocal laser scanning microscope attached to a Leica DM I6000 inverted epifluorescence microscope with a 63× glycerol lens. Videos were recorded at 1 frame/min and were exported from Volocity as QuickTime movies using the Sornson3 video compressor to play at 6 frames/s. 3-D reconstruction was performed in Volocity before export. In order to investigate leukocyte recruitment in the absence of H_2_O_2_, larvae were pre-incubated in 0.3% Danieau's solution supplemented with 100 µM DPI (Sigma) in 1% DMSO, 60 min before and throughout the imaging period. DDC (Sigma) was used at 100 µM, and again embryos were treated 60 min before and throughout the imaging period.

### Imaging of H_2_O_2_ Generation

H_2_O_2_ imaging using a live cell fluorescein dye was performed as previously described [26]; in brief, 4-dpf RAS^+^ larvae and their control siblings were loaded for 60 min with 50 µM acetyl-pentafluorobenzene sulphonyl fluorescein (Calbiochem Merck) in 1% DMSO in 0.3% Danieau's solution and imaged as above. To test the dye efficiency, control caudal fin incisional wounds were imaged in WT larvae.

### Immuno-Spin Trapping

Zebrafish larvae with V12RASeGFP^+^cells or control eGFP^+^ cells, and V12RASeGFP^+^/DUOX morphants were incubated in Danieau's medium containing 200 mM DMPO (Enzo Life Sciences) from 48 hpf to 56 hpf, then fixed in 4% PFA overnight at 4°C. Larvae were subsequently processed for immunostaining using rabbit anti-DMPO (1∶500) (ALX-210-530-R050, Enzo Life Sciences), as described above for standard primary antibodies, in order to reveal ROS exposure. Cy3-conjugated secondary antibody (Jackson Laboratory) was used to reveal the primary antibody.

### Time-Lapse Movie Quantification, Cell Tracking, and Cell Speed Measurements

All the time-lapse movie quantification and tracking analysis was done using Volocity 4.0.4 or 5.0.2 (PerkinElmer-Improvision). Individual LysC:DsRed^+^ cells were identified using automatic “find objects use intensity” and “color the object” functions in Volocity for all the time points to generate a “footprint map.” The final “footprint map” was manually corrected by de-selecting the green fluorescent RAS^+^ cells. Cell size was calculated using the “find objects use intensity” function in Volocity. Cell tracks were generated using the “manual track the object” function in Volocity, which automatically calculates the average cell speed and MI (MI = *D*/*T*, where *D* is the shortest linear distance between the start and end point of the migration path and *T* is the total distance traveled by the cell). MI reflects a cell's directionality, with the highest MI = 1, indicating highly directional migration. Each migrating cell was tracked as long as it could be distinguished from other migrating cells and only if it had been present in the movie for more than ten time points. Cell tracks were then overlaid onto individual images from movies. “Retention time” of leukocytes in the region of RAS^+^ cells was defined as the duration of time that a leukocyte exhibited direct contact with a RAS^+^ cell. “Retention time” for a wound response was defined as the duration of time that a LysC:DsRed^+^ cell stayed within the central wound area (Figure 2B insert highlights this zone).

### Clonal Analysis of Transformed Cell Progression after Preventing Immune Cell Interactions

Embryos at the one cell stage were injected with control, DUOX+p53, and pu.1 morpholinos at 0.2–0.8 mM as described above. A complementary pharmacological approach involved incubation with 50 µm of the NOX inhibitor DPI from 36 hpf to 60 hpf—higher concentrations or longer treatment times with this drug disrupt embryo development, and so are not suitable for our clone analysis (data not shown). At 3 dpf for the morphant studies and 60 hpf for the DPI-treated embryos, larvae were fixed in 4% PFA for 1 h at room temperature, washed briefly in PBS, and mounted laterally to count numbers of V12RASeGFP^+^ cells in flank skin by comparison to controls.

### Statistics

All the data were analyzed (Prism 4.1, GraphPad Software) using either an unpaired two-tailed Student's *t* test or Mann Whitney *U* test for comparisons between two groups, and one-way ANOVA with appropriate post-test adjustment for multiple group comparisons.

## Supporting Information

Figure S1 **V12RAS induces pro-inflammatory gene up-regulation in zebrafish larvae.** (A) RT-PCR showing up-regulation of pro-inflammatory genes in V12RAS^+^ larvae at 4dpf compared with their V12RAS^−^ siblings. (B) qPCR showing increased expression of *il1*β and *cxcl1* in 5-dpf hsp:V12RASeGFP larvae compared with WT after both have been heat shocked for 6 h. (C) Fluorescent in situ hybridization of *arginase1* (cyan) combined with L-plastin antibody staining for leukocytes (magenta) and anti-RAS antibody staining for V12RAS^+^ cells (green) in 7-dpf V12RAS^+^ larvae. (D) Anti-TNFα antibody staining (cyan) combined with anti-L-plastin antibody staining for leukocytes (magenta) in 7-dpf larvae with V12RASeGFP^+^ clones (green)—arrowheads indicate TNFα signal inside some of the L-plastin^+^ cells. *, *p*<0.05. Scale bars = 20 µm.
(3.42 MB TIF)

Figure S2 **RT-PCR for DUOX Showing the efficiency of the DUOX splicing block morpholino.** DUOX-morphant-typing primers amplify a 264-bp WT band from the cDNA of control, un-injected embryos, but embryos injected with DUOX morpholino exhibit exon escape, resulting in a 39-bp deletion of mRNA such that DUOX-morphant-typing primers amplify a smaller band from cDNA of DUOX morphant embryos. This knockdown is full at 3 dpf and retained until 5 dpf.
(0.10 MB TIF)

Video S1 **Supporting movies for** **Figure 1B** **.** (A) shows several MPO:GFP*^+^* neutrophils as they are recruited to a mitfa:V12RAS-mitfa:mCherry-expressing clone of melanocytes in the flank skin of a 5-dpf zebrafish larva (Figure 1Bi). The movie was taken at one frame every 90 s. Black pigmentation quenches much of the mCherry signal (red). The DIC channel is overlayed to show the outline of the V12RAS^+^ clone. The yellow arrowhead in the movie indicates a small fragment of a V12RAS^+^ melanocyte as it is engulfed by an MPO:GFP^+^ neutrophil; later this fragment is seen as a black phagosome inside the neutrophil cell body. (B) is the same sequence as in (A) but without the DIC channel. (C) is a control larva with melanoblasts expressing mCherry alone; in this example, several MPO:GFP^+^ cells come close to the mCherry-expressing cells but are not drawn to the clone.
(10.22 MB MOV)

Video S2 **Supporting movies for** **Figure 1C** **.** (A) shows a control larva showing a single LysC:DsRed^+^ leukocyte passing in the neighborhood of, but not attracted to, GAP43-eGFP-expressing cells. By contrast, (B) shows several LysC:DsRed^+^ leukocytes as they are recruited to a group of v-Src^+^ positive cells in the skin layer of a 3-dpf zebrafish larva that has been injected with pBR-Tol2-UAS-GAP43-eGFP-SC-v-Src with pCS2-Gal4FF and Tol2 capped RNA (Figure 1Ci).
(4.95 MB MOV)

Video S3 **Supporting movies for** **Figure 2** **.** (A) shows the flank skin region of a typical *Tg(kita:GalTA4, UAS:eGFP, LysC:DsRed)* larva (control) at 4 dpf. Green cells are kita:GalTA4/UAS:eGFP^+^ mucus-secreting cells located within the flank skin epidermis. Very few LysC:DsRed^+^ cells were present in the movie, and none were recruited towards GFP^+^ cells. (B) LysC:DsRed^+^ recruitment toward a laser wound (indicated with a yellow line) made within the region of skin shown in Figure 2. The movie commences at 7 h post-wounding and extends for 80 min. (C). is a typical movie from the flank skin region of a *Tg(kita:GalTA4, UAS:V12RASeGFP, LysC:DsRed)* larva at 4 dpf. Green cells are V12RAS^+^ mucus-secreting cells, and here we see LysC:DsRed ^+^ leukocytes actively migrating through the territory of these cells.
(5.30 MB MOV)

Video S4 **Supporting time-lapse movies for** **Figure 3** **.** This panel of movies shows the progressive resolution of LysC:DsRed^+^ leukocytes after recruitment to wound. (A) is early post-wounding (0.5 h post-wounding); (B) is a medium stage wound (5 h post-wounding); (C) is a late stage wound (9 h post-wounding). There are more LysC:DsRed^+^ cells recruited to the wound initially, and they remain at the wound site for a longer period of time. Leukocyte recruitment towards the wound reduces over time during the medium stage and finally resolves at the late stage.
(2.46 MB MOV)

Video S5 **Supporting movies for** **Figure 3** **.** (A) is a time-lapse movie made from a Fli:eGFP/kita:GalTA4/UAS:V12RASeGFP^+^ larva that shows the earliest recognition of V12RAS-transformed cells by Fli:eGFP^+^ leukocytes at the initial stage of transformed cell expansion. Green arrows indicate V12RAS^+^ mucus-secreting cells. These cells express V12RASeGFP and so GFP appears membrane-located in these cells. Leukocytes are motile and labeled by Fli:eGFP, and the GFP signal in these cells has a pale cytoplasmic distribution. (B) shows an example of how LysC:DsRed^+^ leukocytes respond to V12RAS^+^ clones at their “early developmental” stage. (C) shows an example of how LysC:DsRed^+^ cells respond to a “late developmental” stage V12RAS^+^ clone. Note how LysC:DsRed^+^ cells stay with the clone for a prolonged period.
(9.91 MB MOV)

Video S6 **Supporting time-lapse movies for** **Figure 4** **.** This panel of movies shows the representative cell morphology and migration behavior of four leukocyte subtypes. (A) provides a comparison of the morphology and migration behavior of the Fli^+^LysC^−^MPO^low^ (pink arrow) and the Fli^+^LysC^high^MPO^high^ (red arrow) subtypes. (B) provides a comparison of the morphology and migration behavior of the Fli^+^LysC^high^MPO^high^ (red arrow) and Fli^+^LysC^low^MPO^−^ (blue arrow) subtypes. (C) provides a comparison of the cell morphology and migration behavior of the Fli^+^LysC^high^MPO^high^ (red arrow) and Fli^+^LysC^−^MPO^−^ (green arrow) subtypes. Color-coded arrows were used in the movies to help identify each subtype as they appear in the movie. Image sizes were re-scaled to fit in the same frame.
(6.40 MB MOV)

Video S7 **Supporting time-lapse movies for** **Figure 4** **.** (A) *Tg(fli:eGFP, LysC:DsRed)* larvae reveal all the leukocyte subtypes as they respond to a wound (outlined in white) made to the ventral fin. (B) A *Tg(kita:GalTA4, UAS:V12RASeGFP, fli:eGFP, LysC:DsRed)* larva illustrates how all four leukocyte subtypes respond to V12RAS^+^ clones (green). Color-coded arrows in the movies indicate representative cells for each subtype of leukocyte: pink arrow indicates Fli^+^LysC^−^MPO^low^ cells (macrophages), green arrow indicates Fli^+^LysC^−^MPO^−^, blue arrow indicates Fli^+^LysC^low^MPO^−^, and red arrow indicates Fli^+^LysC^high^MPO^high^ (neutrophils).
(6.70 MB MOV)

Video S8 **Supporting time-lapse movie for** **Figure 5** **.** This movie of a *Tg(kita:GalTA4, UAS:V12RASeGFP, fli:eGFP, LysC:DsRed)* larva shows early interactions between V12RAS^+^ cells and three leukocytes. A single V12RAS^+^ cell and a Fli^+^LysC^−^MPO^low^ (macrophage), and two Fli^+^LysC^high^MPO^high^ (neutrophils) extend protrusions toward each other. Red arrowhead points to the tail of a neutrophil, and purple arrowheads indicate macrophage dendritic morphology.
(1.36 MB MOV)

Video S9 **Supporting time-lapse movie for** **Figure 5** **.** In a *Tg(kita:GalTA4, UAS:V12RASeGFP, fli:eGFP, LysC:DsRed)* larva, we see leukocytes as they form tethers with V12RAS^+^ (green) cells. The first tether becomes apparent (yellow arrow) as a neutrophil (red) draws back from the V12RAS^+^ cell. A very pale green macrophage is indicated by a purple arrow, and it, too, leads to a V12RAS^+^-cell-derived tether extending from the transformed cell. Occasionally, leukocytes can be drawn back to the V12RAS^+^ cell, apparently along the tether that links them (second yellow arrow).
(9.63 MB MOV)

Video S10 **Supporting movie for** **Figure 5** **.** A 3-D reconstructed movie showing the engulfment behavior of a LysC:DsRed-labeled neutrophil, as it twice visits a clump of two V12RAS^+^ cells and “nibbles” pieces from them.
(6.73 MB MOV)

Video S11 **Supporting movie for** **Figure 6** **.** A time-lapse movie of a H_2_O_2_ dye (acetyl-pentafluorobenzene sulphonyl fluorescein)–loaded V12RAS^+^ larva with a cluster of V12RAS^+^ (green) cells. At 11 min the dye reports a pale green pulse (green arrow) of H_2_O_2_, and within 5 min, the first neutrophil arrives, and others subsequently appear to focus on this zone of the transformed cells.
(3.65 MB MOV)

Video S12 **Supporting movies for** **Figure 6** **.** A panel of movies showing recruitment of LysC:DsRed^+^ leukocytes to similar flank regions of V12RAS^+^ larvae that are either (A) control, untreated, (B) DPI-treated, (C) DDC-treated, or (D) DUOX morphant.
(3.30 MB MOV)

Video S13 **Supporting movies for** **Figure 7** **.** (A) is a control movie of a LysC:DsRed^+^ embryo injected with hsp:V12RASeGFP. (B) and (C) show LysC:DsRed^+^ cell recruitment in chimeric embryos, corresponding to the footprint images in Figure 7D–7F. (B) shows neutrophils being actively recruited towards DUOX morphant V12RAS^+^ cells in a WT host environment. (C) shows neutrophils similarly being drawn to WT V12RAS^+^ cells in a DUOX morphant host environment.
(2.51 MB MOV)

Video S14 **Supporting negative control movie for data presented in** **Figure 7** **.** This movie shows that cells expressing eGFP alone do not recruit leukocytes when transplanted into LysC:DsRed^+^ larvae.
(1.82 MB MOV)

## Abbreviations

DDC: diethyldithiocarbamate
DMPO: 5,5-dimethyl-l-pyrroline N-oxide
DPI: diphenyleneiodonium chloride
hpf: hours post-fertilization
MI: meandering index
NOX: NADPH oxidase
qPCR: quantitative PCR
RT-PCR: reverse transcription PCR
SOD: superoxide dismutase
WT: wild-type
