# Correction: Live Imaging of Innate Immune Cell Sensing of Transformed Cells in Zebrafish Larvae: Parallels between Tumor Initiation and Wound Inflammation

**DOI:** 10.1371/journal.pbio.1002377

**Published:** 2016-02-11

**Authors:** Yi Feng, Cristina Santoriello, Marina Mione, Adam Hurlstone, Paul Martin

The authors would like to clarify several issues recently raised by the *PLOS Biology* editors.

In Fig 1, it was not clearly indicated that the images of larvae in D and E had been constructed by tiling several micrographs together. The authors have provided a corrected figure and legend, which now clearly indicate the tiling borders with dotted lines.

**Fig 1:**
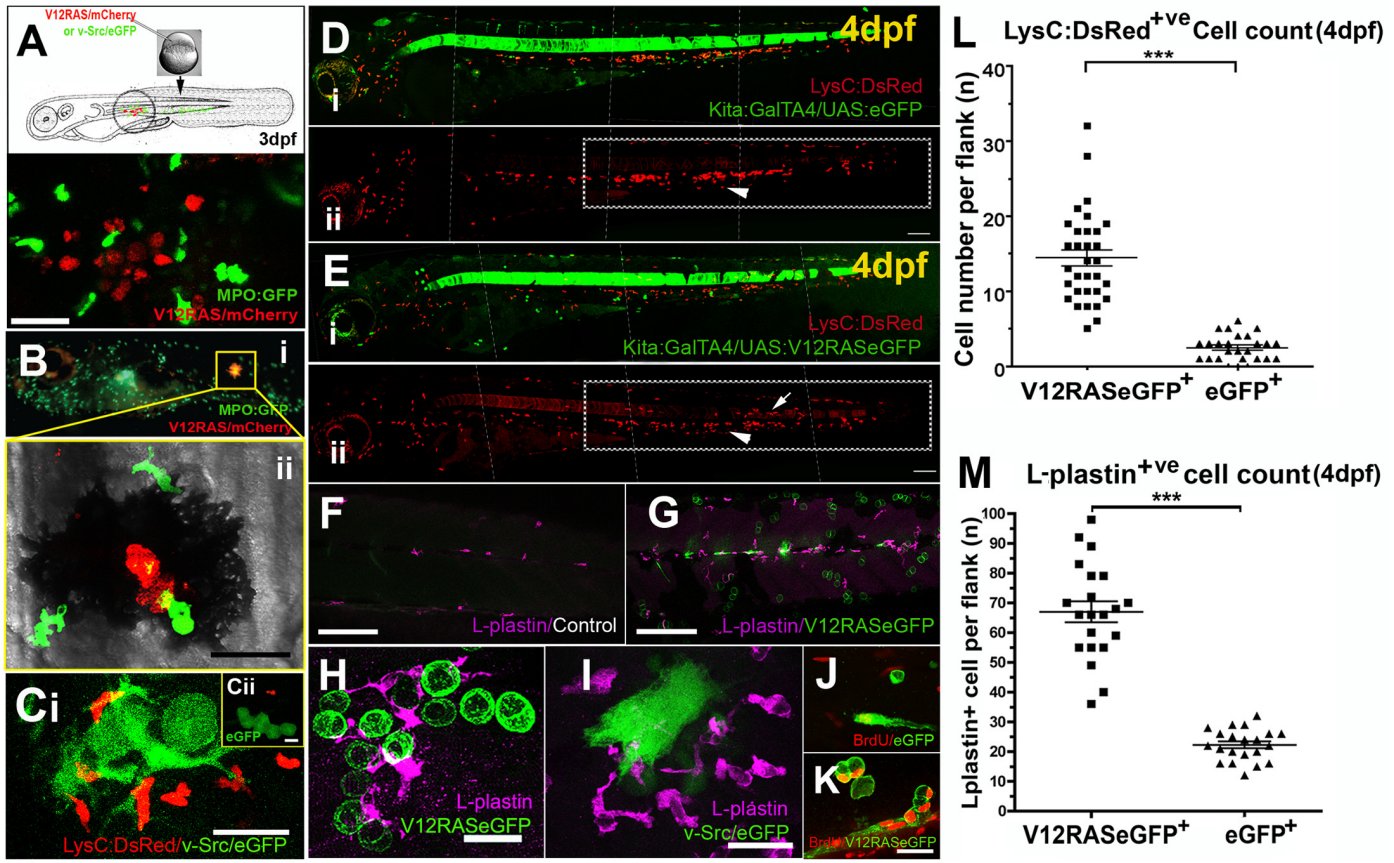
Activation of leukocytes in zebrafish larvae by oncogene transformed cells. (A) Schematic of the procedure for transient induction of V12RAS/v-Src in embryos that also have fluorescently-tagged neutrophils, with an example of a V12RAS^+^ melanoblast clone (red) in a 3dpf larva also expressing eGFP (green) in neutrophils. (B) (i) A 5dpf MPO:GFP larva with a V12RAS^+^ clone (red). (ii) High magnification view of the inset in (B) (i), which is a single image from a time-lapse movie (Video S1A) of GFP-tagged neutrophils actively interacting with a V12RAS^+^ clone; note that most of the red fluorescent signal is quenched by melanocyte pigment. (C) (i) A single image from a time-lapse movie showing LysC:DsRed^+^ cells recruited v-Src^+^ (green) cells in a 3dpf larva (Video S2B) (ii) An equivalent image from a time-lapse movie showing no recruitment of LysC:DsRed^+^ cells to GAP43-eGFP expressing cells in a control larva (Video S2A). (D) (i) Low magnification, two-channel, lateral view of a control Tg (*kita*:*GalTA4; UAS*:*eGFP; LysC*:*DsRed*) larva at 4dpf. (ii) Single channel view to highlight only the DsRed-tagged leukocytes. The box highlights these cells located within the caudal hematopoietic tissue. (E) As for D but of a Tg (*kita*:*GalTA4; UAS*:*V12RASeGFP; LysC*:*DsRed*) larva at 4 dpf. The boxed zone in E (ii) indicates how the LysC:DsRed^+^ cells have largely dispersed from the caudal hematopoietic tissue into the flank skin. (F) A confocal Z-stack projection of the flank of a control larva in the trunk region; (G) Equivalent image to E but of a V12RAS+ larva; both are stained with the anti-L-plastin antibody (magenta). (H) A high magnification view of a larva similar to that in (F), illustrating the association of L-plastin^+^ cells (magenta) with V12RAS^+^ cells (green). (I) A similar larvae to that in H, but with v-Src expressing cells (green) (J) Anti-BrdU (red) immunostaining of control mucus secreting cells (green). (K) Anti-BrdU (red) immunostaining of V12RAS^+^ mucus secreting cells (green). (L) Quantification of numbers of LysC:DsRed^+^ cells present in the skin of the trunk in the region indicated by boxes in (D) and (E). (M) Quantification of the number of L-plastin^+^ cells present in the trunk epidermis in regions indicated in (F) and (G). *** p<0.001; Larval images in (D) and (E) have been “tiled” together from several micrographs and the tiling borders are indicated with white dotted lines. Scale bars: (A) = 48 μm; (B) = 24 μm; (C) = 20 μm; (D, E, F, G) = 150 μm; (I, J, K) = 16 μm.

In Fig 5D and 5E, there are a grey tonal “floors” at the bottom of the 3D reconstructions which is an “effect” of the Velocity software used to generate these images. The figure legend has been revised in order to clearly explain this.

**Fig 5:**
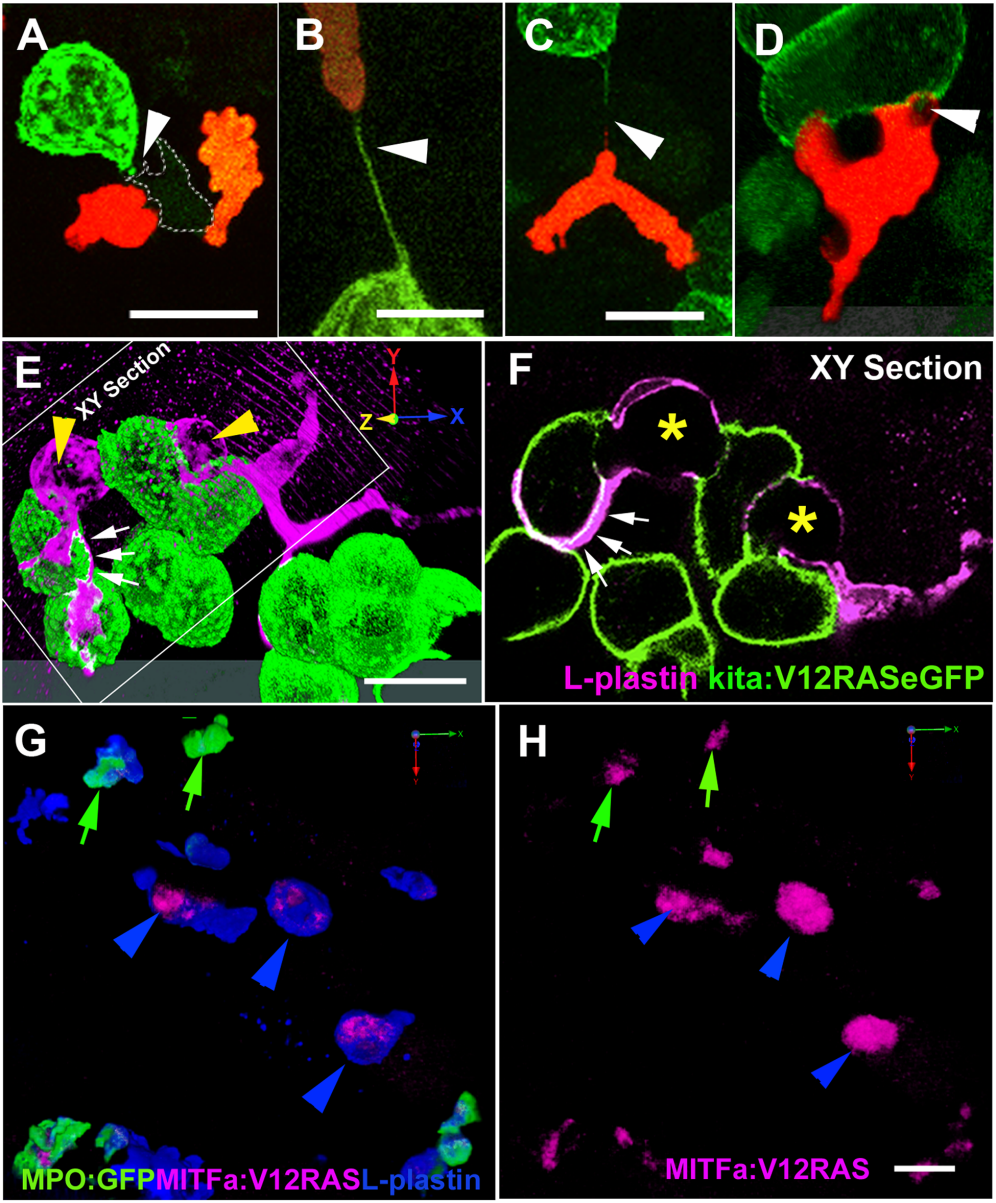
Dynamic interactions between leukocytes and V12RAS^+^ transformed cells. (A) A confocal image of a LysC:DsRed^+^ (red) cell and a macrophage (pale green with dotted line outline) interacting with a V12RAS^+^ cell (green) (Video S8). (B, C) Images showing LysC:DsRed^+^ leukocytes establishing tethers with V12RAS^+^ cells, as observed in Tg(*Fli*:*GFP; LysC*:*DsRed; kita*:*GalTA4; UAS*:*V12RASeGFP*) larvae: (B) shows a tether entirely composed of V12RAS cytoplasm (green) (Video S9); (C) shows a chimeric tether composed of both V12RAS (green) and LysC:DsRed^+^ (red) cytoplasm. (D) A single image from a 3D movie (Video S10), of a neutrophil as it glides over and engulfs pieces of a V12RAS^+^ cell; (E) 3D reconstruction of a V12RAS^+^ cell clump (green cells) showing two macrophages (L-plastin^+^, magenta staining–yellow arrowheads) as they deform to engulf individual V12RAS^+^ cells. Small white arrows and dotted outline indicates lamellipodial protrusions extending over another pair of transformed cells. (F) A single focal plane (corresponding to that indicated in [E]) of the same pair of macrophages, showing V12RAS^+^ cell shaped phagosomes (asterisks), within them. One macrophage has partially enveloped another V12RAS^+^ cell with a thin lamellipodial extension (indicated by small white arrows). (G) 3D reconstruction of an anti-L-plastin stained mitfa:V12RAS-mitfa:mCherry injected Tg(*BACMPO*:*eGFP*) ^i114^ larval flank region, showing the presence of mCherry^+^ (red) debris within L-plastin^+^MPO^-^ cells. (H) Image as in (G) but with single channel to highlight the mCherry^+^ V12RAS^+^ debris; green arrows indicate co-localization within L-plastin^+^ MPO^+^ neutrophils; blue arrowheads indicate co-localization within L-plastin^+^ MPO^-^ macrophages. Note that the larger clumps of debris all reside within macrophages. The grey tonal bars/domains at the bottom of (D) and (E) are the “floor” effect offered by Velocity software when generating a 3D reconstruction from a “Z-stack” of optical sections. Scale bars = 8 μm.

The published version of S1A Fig has duplicated lanes for *tnfα* and *cxcl1*. The authors have provided a corrected version of the figure here, and note that the figure legend remains unchanged. The editors have verified the data underlying the corrected panels and are satisfied that these continue to uphold the conclusions from this figure.

## Supporting Information

S1 Fig V12RAS induces pro-inflammatory gene up-regulation in zebrafish larvae. (A) RT-PCR showing up-regulation of pro-inflammatory genes in V12RAS+ larvae at 4dpf compared with their V12RAS− siblings. (B) qPCR showing increased expression of *il1β* and *cxcl1* in 5-dpf hsp:V12RASeGFP larvae compared with WT after both have been heat shocked for 6 h. (C) Fluorescent in situ hybridization of arginase1 (cyan) combined with L-plastin antibody staining for leukocytes (magenta) and anti-RAS antibody staining for V12RAS+ cells (green) in 7-dpf V12RAS+ larvae. (D) Anti-TNFα antibody staining (cyan) combined with anti-L-plastin antibody staining for leukocytes (magenta) in 7-dpf larvae with V12RASeGFP+ clones (green)—arrowheads indicate TNFα signal inside some of the L-plastin+ cells. *, p<0.05. Scale bars = 20 μm.
(TIF)
