# Supplementary figures and images for: Live Imaging of Innate Immune Cell Sensing of Transformed Cells in Zebrafish Larvae: Parallels between Tumor Initiation and Wound Inflammation
Source: PLoS Biol. 2010 Dec 14;8(12):e1000562. doi: 10.1371/journal.pbio.1000562 (PMC3001901; doi:10.1371/journal.pbio.1000562)

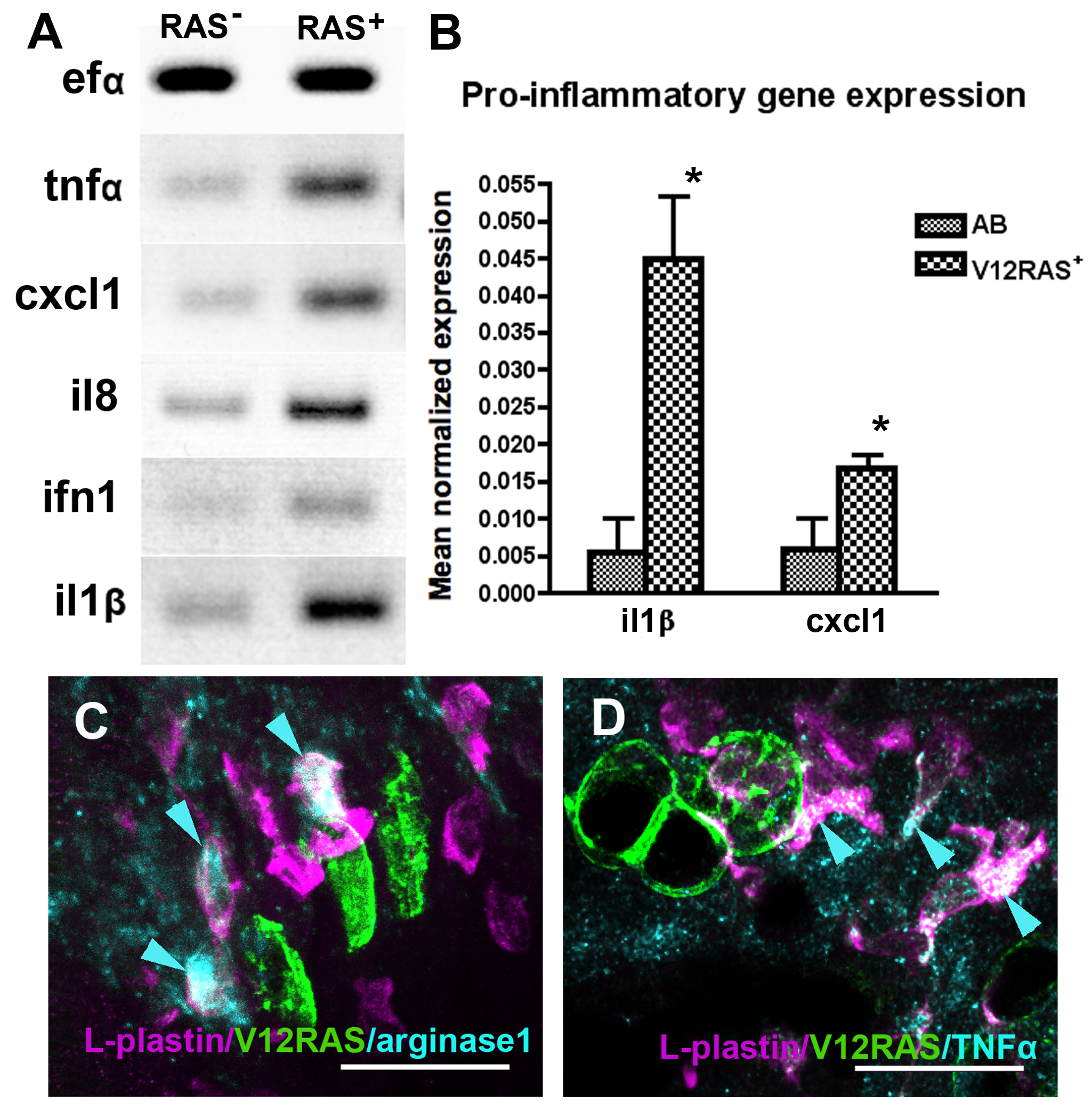

Supplement: Figure S1 — V12RAS induces pro-inflammatory gene up-regulation in zebrafish larvae. (A) RT-PCR showing up-regulation of pro-inflammatory genes in V12RAS+ larvae at 4dpf compared with their V12RAS− siblings. (B) qPCR showing increased expression of il1β and cxcl1 in 5-dpf hsp:V12RASeGFP larvae compared with WT after both have been heat shocked for 6 h. (C) Fluorescent in situ hybridization of arginase1 (cyan) combined with L-plastin antibody staining for leukocytes (magenta) and anti-RAS antibody staining for V12RAS+ cells (green) in 7-dpf V12RAS+ larvae. (D) Anti-TNFα antibody staining (cyan) combined with anti-L-plastin antibody staining for leukocytes (magenta) in 7-dpf larvae with V12RASeGFP+ clones (green)—arrowheads indicate TNFα signal inside some of the L-plastin+ cells. *, p<0.05. Scale bars = 20 µm. (3.42 MB TIF) [file pbio.1000562.s001.tif]

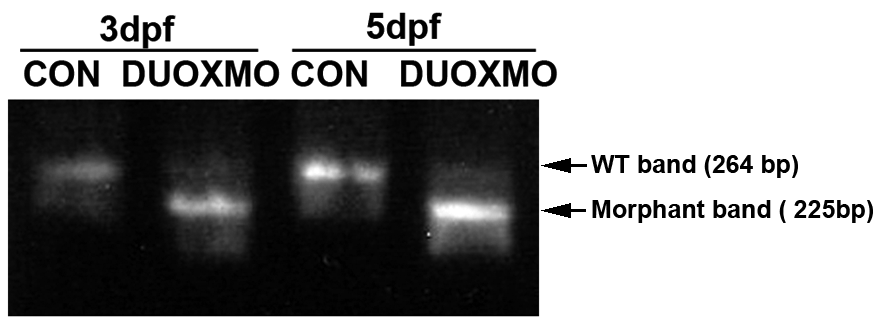

Supplement: Figure S2 — RT-PCR for DUOX Showing the efficiency of the DUOX splicing block morpholino. DUOX-morphant-typing primers amplify a 264-bp WT band from the cDNA of control, un-injected embryos, but embryos injected with DUOX morpholino exhibit exon escape, resulting in a 39-bp deletion of mRNA such that DUOX-morphant-typing primers amplify a smaller band from cDNA of DUOX morphant embryos. This knockdown is full at 3 dpf and retained until 5 dpf. (0.10 MB TIF) [file pbio.1000562.s002.tif]
