# Supplementary figures and images for: Correction: Live Imaging of Innate Immune Cell Sensing of Transformed Cells in Zebrafish Larvae: Parallels between Tumor Initiation and Wound Inflammation
Source: PLoS Biol. 2016 Feb 11;14(2):e1002377. doi: 10.1371/journal.pbio.1002377 (PMC4750939; doi:10.1371/journal.pbio.1002377)

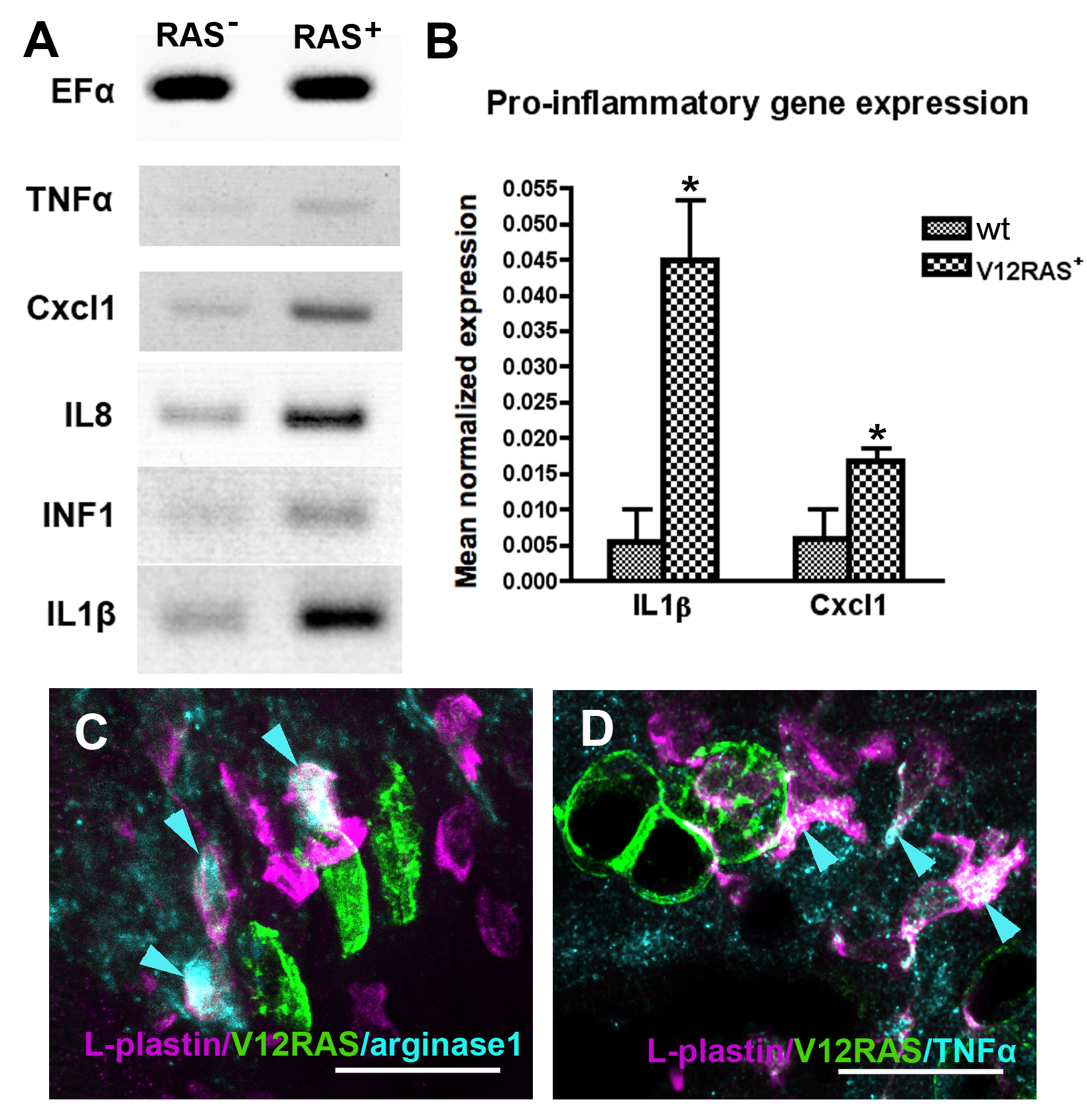

Supplement: S1 Fig — (A) RT-PCR showing up-regulation of pro-inflammatory genes in V12RAS+ larvae at 4dpf compared with their V12RAS− siblings. (B) qPCR showing increased expression of il1β and cxcl1 in 5-dpf hsp:V12RASeGFP larvae compared with WT after both have been heat shocked for 6 h. (C) Fluorescent in situ hybridization of arginase1 (cyan) combined with L-plastin antibody staining for leukocytes (magenta) and anti-RAS antibody staining for V12RAS+ cells (green) in 7-dpf V12RAS+ larvae. (D) Anti-TNFα antibody staining (cyan) combined with anti-L-plastin antibody staining for leukocytes (magenta) in 7-dpf larvae with V12RASeGFP+ clones (green)—arrowheads indicate TNFα signal inside some of the L-plastin+ cells. *, p<0.05. Scale bars = 20 μm. (TIF) [file pbio.1002377.s001.tif]
